# A proposed core curriculum for dental English education in Japan

**DOI:** 10.1186/s12909-014-0239-4

**Published:** 2014-11-18

**Authors:** Omar MM Rodis, Edward Barroga, J Patrick Barron, James Hobbs, Jayanetti A Jayawardena, Ikuo Kageyama, Bukasa Kalubi, Clive Langham, Yoshizo Matsuka, Yoichiro Miyake, Naoko Seki, Hiroko Oka, Martin Peters, Yo Shibata, Roxana Stegaroiu, Kazuyoshi Suzuki, Shigeru Takahashi, Hironori Tsuchiya, Toshiko Yoshida, Katsuhiko Yoshimoto

**Affiliations:** Institute of Health Biosciences Support Office of Frontier Oral Science, International Exchange and Collaboration, The University of Tokushima Graduate School, 3-18-15 Kuramoto-cho, Tokushima City, 770-8504 Japan; Department of International Medical Communications, Tokyo Medical University, Tokyo, Japan; Department of Foreign Languages, Iwate Medical University, Iwate, Japan; Department of General Education, Tsurumi University School of Dental Medicine, Kanagawa, Japan; Department of Anatomy, The Nippon Dental University School of Life Dentistry, Niigata, Japan; Nihon University School of Dentistry, Tokyo, Japan; Dental Education Development Section, Graduate School of Medical and Dental Sciences, Tokyo Medical and Dental University, Tokyo, Japan; Department of International Collaboration Development for Dentistry, Institute of Biomedical & Health Sciences, Hiroshima University, Hiroshima, Japan; Medical English Section, Kanagawa Dental University, Kanagawa, Japan; Division of Biomaterials and Engineering, Department of Conservative Dentistry, Showa University School of Dentistry, Tokyo, Japan; Department of Oral Health and Welfare, Niigata University Graduate School of Medical and Dental Sciences, Niigata, Japan; Department of Endodontics, School of Dentistry, Aichi Gakuin University, Nagoya, Japan; Department of Oral Functional Anatomy, Hokkaido University Graduate School of Dental Medicine, Hokkaido, Japan; Department of Dental Basic Education, Asahi University School of Dentistry, Gifu, Japan; Center for the Development of Medical and Health Care Education (Dental Education), Okayama University, Okayama, Japan

**Keywords:** Health care English, Dental English, Harmonized education, Core curriculum, Japan

## Abstract

**Background:**

Globalization of the professions has become a necessity among schools and universities across the world. It has affected the medical and dental professions in terms of curriculum design and student and patient needs. In Japan, where medicine and dentistry are taught mainly in the Japanese language, profession-based courses in English, known as Medical English and Dental English, have been integrated into the existing curriculum among its 83 medical and 29 dental schools. Unfortunately, there is neither a core curriculum nor a model syllabus for these courses.

**Methods:**

This report is based on a survey, two discussion forums, a workshop, and finally, the drafting of a proposed core curriculum for dental English approved by consensus of the participants from each university.

**Results:**

The core curriculum covers the theoretical aspects, including dental English terms and oral pathologies; and practical aspects, including blended learning and dentist-patient communication. It is divided into modules and is recommended to be offered for at least two semesters.

**Conclusions:**

The core curriculum is expected to guide curriculum developers in schools where dental English courses are yet to be offered or are still in their early development. It may also serve as a model curriculum to medical and dental schools in countries in Asia, Europe, Africa, and Central and South America, where English is not the medium of instruction.

**Electronic supplementary material:**

The online version of this article (doi:10.1186/s12909-014-0239-4) contains supplementary material, which is available to authorized users.

## Background

**Figure 1:**
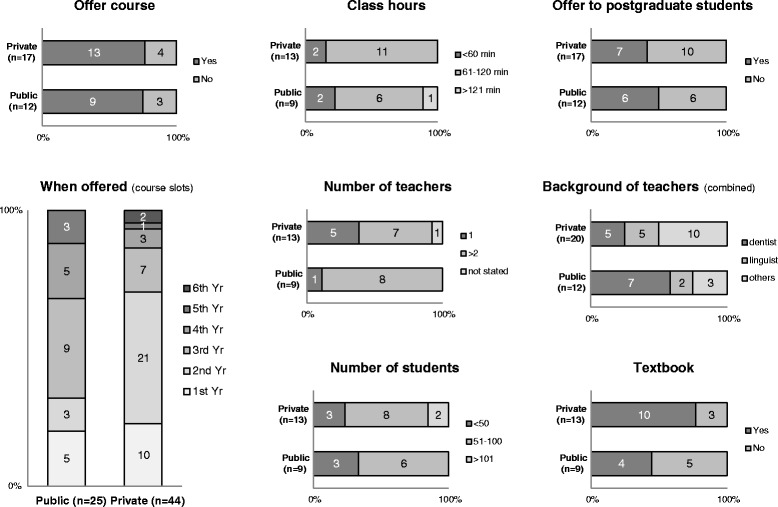
**Status of dental English courses taught in Japan’s 29 dental schools (as of March 2011).**

Medical and dental schools worldwide have started discussing and even implementing changes, reviews, and development of curricula to address the ever-changing needs of society [1-5]. In Japan, where medicine and dentistry are taught purely in the Japanese language, curriculum change and development is slowly being geared towards globalizing the profession. In 2003, the Ministry of Education, Culture, Sports, Science and Technology presented national guidelines, which aimed to improve the quality of English education in Japan and produce citizens who can function effectively and be competitive in the global society [6,7]. The guidelines called for a shift from repetition and mastering grammar toward an emphasis on functional, communication-oriented teaching and the development of listening and speaking skills. This has led to higher educational reforms among universities aiming to produce unique and marketable education programs, locally and internationally. English became integrated into the curricula of different professional fields. In dentistry, such courses are variously known in dental schools as “Dental English”, “English for Dentistry”, “English for Dental Medicine”, “*Shigakubu Eigo*”, “*Shigaku Eigo*” or “*Shika Eigo*”. These courses aim to teach English dental terminology and present conversational situations commonly used in the dental setting. Unfortunately, not all of Japan’s 29 dental schools offer such courses (Figure 1 and in Unit 7 of Additional file 1) [8,9]. The core curriculum should address the ever-changing needs and concerns of the profession, and should include courses that will develop vocabulary, inter-profession and patient-dentist communication and student-motivation. This report summarizes the input and feedback of participants representing their respective dental schools during the first-ever discussion forum on dental English education in Japan (full report is available as an Additional file 1). It presents a foundation for a common core curriculum for dental English courses by highlighting the current situation and future needs of students, teachers, patients, and dental schools. Notably, the views or opinions in this report are those of the participants and experts convened for the discussion forum and workshop, and are not necessarily the official views or opinions of their respective schools.

The aim of this report is to provide teachers and education officials with a core curriculum or ideas on which to base their further developments of English language courses appropriate to the needs and goals of their schools and students.

## Methods

Prior to the start of the study, a grant proposal to develop a core curriculum for dental English courses was submitted to the Japan Society for the Promotion of Science, an independent administrative institution in Japan, established for the purpose of contributing to the advancement of science in all fields of the natural and social sciences and the humanities, in October 2010. In February 2011, a survey questionnaire was sent to the Academic Affairs Office of Japan’s 29 dental schools. The 4-page survey questionnaire contained a cover letter explaining the background and purpose and was written in English with Japanese subtitles. Questions of the 8-item survey included: 1) Do you offer a dental English course? 2) When is it offered? 3) What is the length of classes? 4) What is the average number of students? 5) What is the number of teachers? 6) What is the background of teachers 7) Do you use designated textbooks? and 8) Do you offer elective dental English education to postgraduate students? After obtaining a 100% response rate within 1 month, the results were collated, tabulated and reported back to the 29 dental schools (Please see Additional file 1). After the 3-year 5 million yen proposal was approved in May 2011, preparations began for organizing a discussion forum to assess the current situation of dental English education among Japan’s 29 dental schools. An invitation letter was sent to the Academic Affairs Office of all dental schools. On August 28, 2011, the first ever discussion forum concerning dental English was held in Okayama, Japan. The aims were to initiate a gathering of teachers of dental English courses from Japan’s 29 dental schools, to discuss how to develop and implement a core curriculum for the course, and to organize a support group for teachers of the course. Discussions on the background and summary of the current status of dental English among Japan’s dental schools and student competency as well as on syllabus implementation and teaching/learning methods were held in the morning session. Discussions on strategies of respective teachers on applying English medico-dental terminologies to the undergraduate dental course, agreement on “general instructional objectives (GIO) and specific behavioral objectives (SBO)” of at least 2 or 3 years’ courses, the feasibility of offering the course as a compulsory dental subject, the possibility of offering the course for 6 years, or if not, deciding the most feasible, and the feasibility of including dental English in the National Dental Board Exams were held in the afternoon session. An interim report based on video and audio recordings of the proceedings of the 1^st^ discussion forum was drafted and sent to all the participants. The 2^nd^ meeting was held in Tokyo on June 9, 2012. Its aims were to organize a workshop/seminar, to gain knowledge and receive advice on core curriculum development from invited speakers, and to discuss and decide the contents of the core curriculum. A seminar on English for Medical Purposes education was held in the morning by guests from Tokyo Medical University. A workshop and discussion forum was held in the afternoon to finalize the core curriculum. The 2^nd^ interim report was drafted and sent to the participants. The final report was drafted, approved by consensus, and 1 book-bound copy was sent to all 29 dental schools, 2 copies to the Japan Ministry of Education, and 1 copy to the Japan Dental Association.

## Results

### Curriculum framework

**Figure 2:**
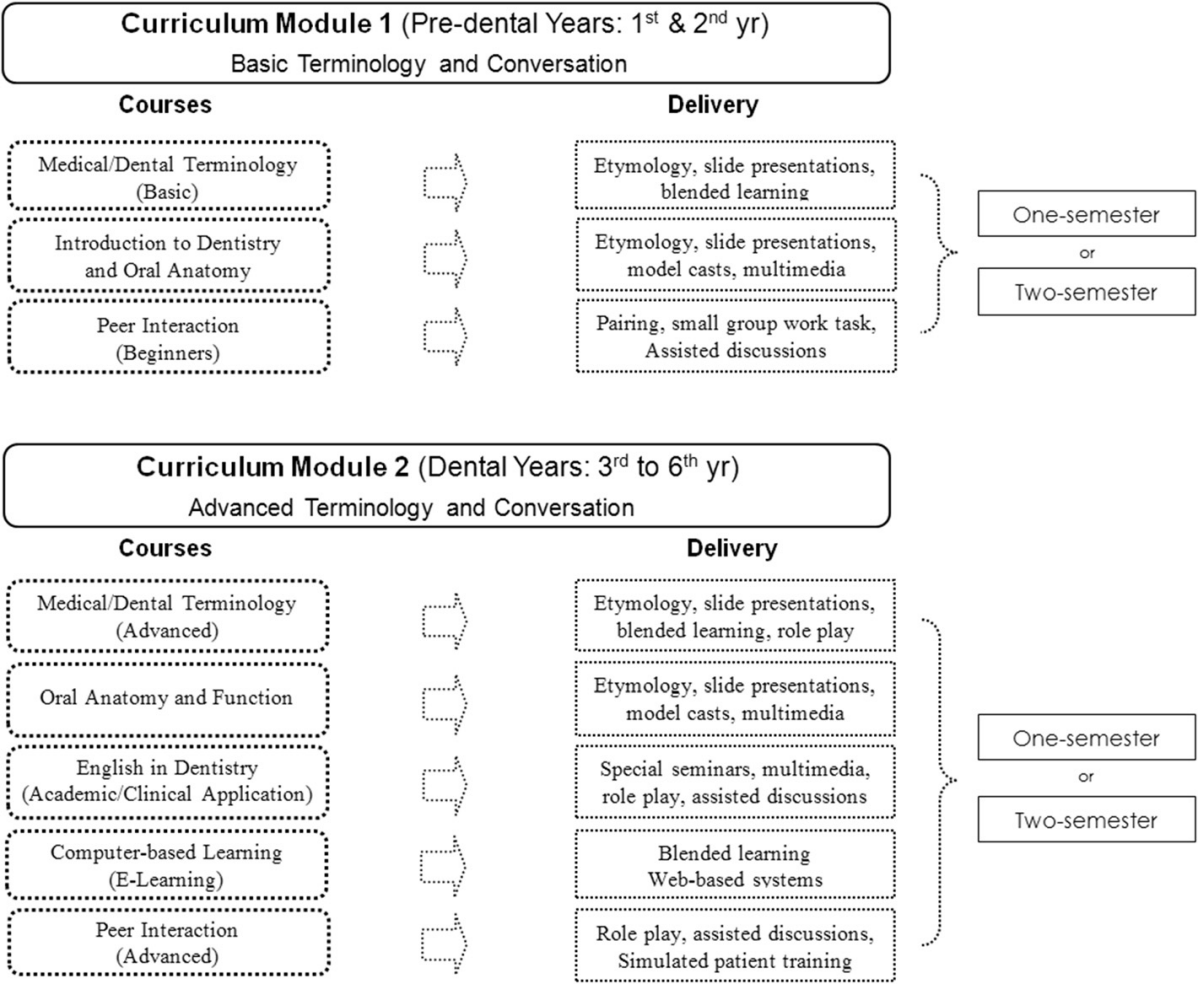
**The curriculum framework for the proposed dental English core curriculum.**

The proposed core curriculum addresses the challenges of globalization of the dental profession. It is divided into two modules of didactic and practical courses offered in any of the pre-dental years (from 1^st^ to 2^nd^ year) and in any of the dental years (from 3^rd^ to 6^th^ year) of the six-year dental curriculum (Figure 2). It covers basic and advanced terminology and conversation aimed at improving self-confidence, dentist-patient and inter-professional communication. Courses in general and dental terminology will include etymology (Greek/Latin derivatives) and principles of terminology (prefix/suffix, combining vowels, and using terms in context). Each module can be offered as a one-semester or two-semester course. Thus, each school can modify the proposed core curriculum according to their needs, as long as the same objectives are used.

### Curriculum module 1: basic terminology and conversation

Module 1 (in Figure 2) may be offered in any of the semesters during the pre-dental years, i.e. from the 1^st^ year to the 2^nd^ year. The first 2 years of the 6-year dental curriculum mainly cover the basic sciences and a few orientation courses on dentistry. Module 1 contains courses covering common words used in daily interaction with patients, introduction to dentistry, basic medico-dental terms and their etymology, and simple interactive exercises to motivate students concerning the importance of professional globalization.

### General instructional objective

Students will learn how to communicate with English-speaking patients on basic topics of routine dental consultation and treatment.

### Specific behavioral objectives

Students will be able to say and respond to basic dental terms and phrases.

Students will be able to understand what the patient says in English.

Students will be able to ask their patients about their medical and dental conditions as well as explain dental procedures in English.

Students will be able to access and use fact sheets for native speakers at websites, for example, international dental associations, and international journals.

### Medical and dental terminology (basic)

#### Course description

The course covers basic medical and dental terms commonly used in general situations. It includes casual and formal ways of greeting, speaking and listening for professional duties, meetings or activities. Additionally, knowledge of common general terms such as used in the dental clinic, reception, and making appointments, as well as medical terms such as fever, cold, diabetes is included as it is an integral part of history taking, diagnosis, treatment and patient-dentist or inter-professional interaction. A significant part of the course emphasizes the etymology of commonly used terms with a focus on overall meaning.

### Delivery

Teaching methods include blended learning, presentations and etymology. Blended learning emphasizes learning through the use of technology and face-to-face interaction. Although gaining popularity, there are considerations that have to be met in blended learning. This approach emphasizes learning through the use of technology such as access to online resources, communication via social media or interaction with distance learners in other classrooms, use of audio-video files, and face-to-face interaction. Others include restrictions in availability of resources, the competency and nature or location of the school and students. Slideshow presentations should be customized to the level of understanding of the students. This will be an effective tool in enhancing better understanding and memory retention of new topics. Subtitles on slides with new or technical terminology should be used to augment student comprehension as visual aids. The medium of instruction should be English followed by a very brief translation in Japanese to emphasize the conveyed message particularly for new terms. English must be pronounced clearly and slowly. For technical terms, it is necessary for students to learn the basic principles in terminology structure since medical/dental terminology include Latin or Greek root words, prefixes, suffixes and combining vowels.

### Introduction to dentistry and oral anatomy

#### Course description

The course focuses on the history, scope and the science of dentistry, and the basic nomenclature of the teeth and their supporting structures. Topics include the introduction of dentistry as a science and profession plus the names of the types of teeth and the structures surrounding it.

### Delivery

Teaching methods include word etymology, slideshow presentations and the use of model casts or multimedia. Etymology is important in understanding the meaning of terms commonly used in medicine and dentistry. In addition to etymology, slideshows must contain many pictures or videos of the teeth and their related structures to enhance learning and interest of students. The use of model casts is also one way of enhancing the learner’s cognitive learning through visual input.

### Peer interaction (beginners)

#### Course description

The course focuses on pairing or grouping students to perform specific tasks or roles for a given simple dentist-patient situation. It has been proven that effective interaction with peers becomes a successful and powerful learning method only if students encourage each other to ask or answer questions, explain and express their thoughts. However, the majority of Japanese students feel uneasy about expressing their thoughts in public, and much more so in English. The beginners’ course must therefore introduce to students the importance of improving one’s social skills, which they will need in their future professional interactions, through assisted discussions, peer pairing or small group work tasks. Activities should include situations in casual and formal greetings, group discussions on simple topics of interest, inquisitiveness exercises, gesturing, facial expressions and culture studies, among others.

### Delivery

Almost all conversations, whether they be formal or informal, start with a greeting. The same is applicable when meeting patients or colleagues for the first time, seeing the patients again for recall, self-introduction, and introducing others. Expressions of farewell and taking care of health are equally important. Role playing by pairs consists of situations like meeting someone for the first time, meeting someone again, or introducing your friend to others. Role playing by small groups can be about trying to get to know someone in class you do not know and introducing him/her to the class. Most of the students may already know each other but it is important to emphasize to them that the purpose of the exercise is to practice their English listening and speaking skills.

Assisted discussions may be performed by reading and discussing simple cases in daily living among the group with teacher assistance. Inquisitiveness exercises include having students go around and ask questions of each other or having them give out their self-formulated quizzes to their classmates. This exercise will prepare them in gaining confidence in asking questions in future classes. Lectures and practical exercises on gesturing, facial expressions and culture studies should also be included since these also form an important part of conversation.

### Curriculum module 2: advanced terminology and conversation

Module 2 (in Figure 2) may be offered in any of the semesters during the dental years, which are from the 3^rd^ year to the 6^th^ year. The last 4 years of the 6-year dental curriculum mainly cover didactic and clinical dentistry. Module 2 contains courses covering terms used in the patient- and inter- and intra-professional interaction, etymology of advanced medical and dental terms, and interactive exercises focusing on dental clinical, academic, research and international situations. This will provide students with the basic knowledge needed for self-confidence, self-improvement, dentist-patient communication and inter-professional communication.

### General instructional objective

Students will acquire the basic skills of professional communication.

### Specific behavioral objectives

Students will learn to say and respond to technical (dental) terms and phrases.

Students will create posters on dental topics.

Students will make oral presentations on dental topics.

Students will correspond with other health professionals.

(For example: e-mail, lectures, meetings, *etc.*)

### Medical and dental terminology (advanced)

#### Course description

The course covers more advanced/technical word lists of general, medical and dental terminology and studying in context these terms and sentence structures from different conversational situations. Since most medical terminology and dental terminology are derivatives of the Greek or Latin languages, it is imperative to study etymology, root words, prefix, suffix and combining vowel terminology structure.

### Delivery

Teaching methods include blended learning and etymology. As with Module 1 (in Figure 2), teaching new terms should focus on overall meaning and not on memorization and therefore if the situation or flow of conversation changes, students should be able to understand or rephrase their statements accordingly. This is the reason why teaching terminology should be taught in context, and not just the term per se. Teaching should be supplemented by multimedia (video, audio, slideshow presentations) and if possible, pronunciation lessons and exercises. Knowledge of the etymology of most medical and dental terms will allow students to better understand the principles of terminology than rote learning or memorization. Additionally, teaching terms in context will help students understand the use of the term better. Etymological units can be used in blended learning to change/substitute the terms or meanings so students can improve comprehension. Pronunciation guides or audio clips may be inserted into slideshow presentations together with pictures or videos showing the meaning of the terms or parts of the term and uploaded online for the student’s future reference.

### Oral anatomy and function

#### Course description

The course covers the nomenclature and function of the mouth, teeth and surrounding structures. Having this course in Module 2 (in Figure 2) will, in parallel, augment the student’s knowledge in oral anatomy and function since they will be learning this in the Japanese context as well. Thus, it will be easier for them to inter-relate terminology and function. Since oral anatomy is the basis of topics and situations in the dental profession, it is important for students to have a good foundation and understanding of it upon completion of this course. Topics should therefore include the following: types and structures of teeth, periodontium, upper and lower jaw, oral tissues, eruption patterns, tooth numbering systems, oral health and disease (oral hygiene, dental caries, caries assessment), dental materials (types of materials/instruments, prosthetics) and functions (oral tissues, saliva, chewing, occlusion, speech).

### Delivery

Teaching methods include blended learning, etymology, slideshow presentations, and the use of model casts. Other teaching methods may be also adapted to meet the goals of this course. Information is basically presented through lectures since the course is a basic dental science. Non-dentist teachers can invite guest lecturers who are dentists in case the need arises. Audio-visual aids that include clinical slides, videos, online sources and teaching model casts should be used to support classroom and practical activities.

### English conversation in dentistry (academic/clinical application)

#### Course description

Applied conversations regarding dental situations constitute a major part of this course. The academic segment of the course focuses on communication between and among professionals as well as patients. Future dentists should be able to prepare themselves to meet, interact or collaborate with colleagues from other countries and should continue to aspire for knowledge by reading scientific publications or presenting research at international conferences. The course also includes mentoring on how to read and write scientific papers, improve presentation skills, and foster research collaboration. The clinical segment will focus on patient-dentist communication, which is the verbal and non-verbal process through which a dentist obtains and shares information with a patient; and chair-side conversation exercises on dental consultation and treatment. The knowledge gained from basic and advanced terminology courses is applied in this course.

### Delivery

Applied conversation may be performed by groups through role playing. Since the focus is on dental situations, teachers should prepare a large number of case scenarios beforehand. Cooperation among teachers may facilitate matters. Participants may act within their own groups or with other groups. Case scenarios include a wide variety of situations including self-introduction to previously known and unknown colleagues, presenting a case study, answering inquiries by telephone, interviewing first-visit patients, medical/dental history-taking, scheduling recall visits, chair-side conversations, difficult-to-manage patients and pediatric patients. Students may be tasked to write dialogues for a case scenario they decide on and they can practice it amongst themselves or with other groups. If the English teacher (native/non-native) requires any assistance regarding teaching or preparation on dental-based case scenarios, he may invite a dentist or foreign researchers with a dental background.

### Computer-based learning (E-learning)

#### Course description

Learning in an electronically supported environment is known to help in the implementation of the learning process. E-learning is essentially the computer and network-enabled transfer of skills and knowledge. Content is delivered via smart phones, internet, audio or video files, or through online databases. It can be self-paced or instructor-led and includes media in the form of text, image, video and audio. If resources permit, adequate volumes of multimedia files, questionnaires, multiple-choice quizzes or electronic patient charts should be available for students. Computer-based activities integrated with practical or classroom-based situations have become increasingly popular in higher education and language learning. Teachers may set timeframes for content access and response or make the content freely available throughout the semester. Although the system can be beneficial, the set-up and maintenance of computers and device compatibility may require specialized staff.

### Delivery

Private E-learning platforms in Japan have collaborated with a number of universities in response to their specific academic needs. For instance, a system would include customizable reading, listening and writing exercises on a conversation between a tourist and a local. If the dental school subscribes to such services, teachers should familiarize themselves with their content and applicability. Otherwise, teachers can design their own online tools using platforms that are available for free or those maintained by the university network. Teachers may design online quizzes for students to answer outside class or during their free time and in the same way, return results online. It is also possible for teachers to allow students to access print-outs of upcoming lectures so they will have an idea of the topics before the class. Additionally, an online discussion forum on case reports or a specific topic may be developed for students to prepare them for actual face-to-face discussions during class.

It is also highly recommended that electronic dental chart recording be included, because most medical/dental institutes use electronic charts. Chart information will help students review and apply their knowledge on terminology, medical/dental conditions, tooth nomenclature and numbering, spelling, and writing skills (in diagnosis, prognosis or note-taking).

### Peer interaction (advanced)

#### Course description

Since learning is social, at its best, it develops from interaction with others, as perceptions are shared, information is exchanged, and problems are solved. This course focuses on pairing or grouping students to perform specific tasks or roles for inter/intra-professional communication. The course will facilitate the improvement of critical thinking and discourse concerning dental topics, including dentist-patient and inter/intra-professional interaction through social interaction between their peers. The course aims to improve student’s social behavior outcomes and demonstrable evidence of self-confidence in social interaction. The type of interaction in a second-language class usually depends on the teachers and most teachers would use a mix of activities to develop fluency and accuracy. In fluency-oriented activities, students should be able to speak without much interruption, encouraging them to use as much of their language knowledge as they can. In accuracy-oriented activities, students should be able to focus on the correct usage of grammar or vocabulary, although relaxed communications should be considered more important than correct grammar. Therefore, teachers should prepare a syllabus that would allot more time on peer-interaction activities and special seminars on professional growth such as speaking and presenting skills. Additionally, the school should be able to create a culture of dual language usage within the university not just among students but also among teachers in other departments.

### Delivery

Group-work activities continue to comprise the advanced course. Scenarios on developing optimism, getting into a group, giving and receiving compliments, eliciting opinions, respecting differences, disagreeing politely, and building a positive reputation, are suggested. In succeeding activities, the use of simulated (standardized) patients as part of the course will prepare students in their future dealings with actual English-speaking patients and colleagues. If possible, teachers should enforce an all English policy in the classroom. Teachers can create motivation in the form of interactive activities where the students need to communicate in order to complete a task. For example, Student 1 has the full information of a case study concerning tooth decay and Student 2 needs to fill in the blanks. During their peer-to-peer discussions, it should be a basic and unalterable tenet that discussions should be in English and teachers, teaching assistants, or the students should circulate facilitating or checking the conversations. The final activity of Module 2 should be training for dentist-patient and inter/intra-professional communication using simulated patients. If possible, this should conform with the Japanese objective structured clinical examination (OSCE) standards with English as a means of communication and also universally accepted standards of inter/intra-professional communications and presentations in scientific meetings.

### Learning strategies and evaluation

GIO and SBO in syllabus design are complemented by learning strategy (LS) and evaluation (EV). In simple terms, LS refers to methods that students use to learn. Weinstein and Mayer defined learning strategies broadly as “behaviors and thoughts that a learner engages in during learning” which are “intended to influence the learner’s encoding process” [10]. Later, Mayer specifically defined LS as “behaviors of a learner that are intended to influence how the learner processes information” [11]. Evaluation is the process through which teachers judge the quality of their own work or those of their students. The EVs of students’ progress and acquisition of skills may include quizzes, classroom activities, assignments or any achievement towards mastering the objectives of the course. Practical or clinical skills may be evaluated through success in OSCE via role play activities or regular practice in skills laboratory training [12]. Since this core curriculum is intended for use in dental schools with different needs and where English is not the medium of instruction for dentistry, sample syllabi are included in the full report (please see Additional file 1). Teachers must formulate their own LSs and EVs according to the priorities, preferences and situation of their respective schools and those of their students.

## Discussion

Curriculum implementation is the practical application of courses, subjects and activities prescribed to help students acquire knowledge and experience. However, it is not the endpoint and, as such, should be evaluated continuously. Globalization and the ever-changing needs of students, dentists and patients require competent professionals with exceptional vision, knowledge, technical, and communication skills. Future Japanese dentists should be able to readily and confidently provide optimal care and instruction to both Japanese and international patients. They should also be able to see themselves as partners in the international community of dental professionals.

The core curriculum for dental English courses presented serves as a guide to curriculum developers regarding the basic and current needs that require addressing as far as English education in dental schools is concerned. The core curriculum was originally developed based on a collection of teaching techniques and experiences of teachers of the course, the current needs of dental students, and a positive outlook for future Japanese dental students and dentists who are globally competitive. Since teachers of the course(s) are the ultimate facilitators, they are absolutely essential in the implementation process, although they are free to choose or mix various aspects of recommended courses, topics or exercises as appropriately as possible. Core curriculum implementation therefore refers to how the officially designed course of study is translated by the teacher into their own syllabi and delivered to students. This translation process is then evaluated regularly by the teachers or teachers and students for further improving the course.

This paper has some limitations. It is primarily focused on the Japanese dental education world. Furthermore, the entire core curriculum for dental English has yet to be fully implemented by even a single dental school. However, parts of the core curriculum, as well as many of its basic concepts, are being gradually introduced in different dental schools in Japan. We are currently receiving feedback and are preparing a preliminary report on the information we are obtaining from other colleagues from other dental schools.

## Conclusions

Our 1^st^ and 2^nd^ discussion forums, a seminar and a workshop proved to be successful gatherings of educators from different dental schools in Japan and effective for developing the core curriculum for dental English courses. Emphasis was focused on the balance of terminology and dentist-patient communication courses, adaptability, implementation, and evaluation. The authors therefore recommend the following as key points for the overall success of the core curriculum: (1) The core curriculum, if possible, should be implemented over at least two semesters of the 6-year dental curriculum; (2) The core curriculum, after implementation, should be evaluated continually through regular assessment and student feedback; (3) Changes or improvements of the core curriculum should be implemented by consensus among mentoring and teaching staff in a fast and timely manner with further regular and continuous evaluation; (4) The core curriculum may be modified according to the needs of the schools, students and teachers; (5) The core curriculum is not intended to replace an existing or established curriculum but rather to guide curriculum developers in coping with the needs of their respective schools; and (6) The core curriculum is freely available for modifications by anyone in any country where English is not the medium of instruction in dental or medical education or to anyone interested in curriculum development.

## Additional file

Additional file 1:
**Proposed Core Curriculum for Dental English Education (The Full Report).**

## Abbreviations

EV: Evaluation
GIO: General instructional objectives
LS: Learning strategy
OSCE: Objective structured clinical examination
SBO: Specific behavioral objective
