# Supplementary material for: A proposed core curriculum for dental English education in Japan
Source: BMC Med Educ. 2014 Nov 18;14:239. doi: 10.1186/s12909-014-0239-4 (PMC4237740; doi:10.1186/s12909-014-0239-4)
Supplement: Additional file 1: — Proposed Core Curriculum for Dental English Education (The Full Report). [file 12909_2014_239_MOESM1_ESM.pdf]

# PROPOSED CORE CURRICULUM FOR DENTAL ENGLISH EDUCATION

Submitted to the Ministry of Education, Culture, Sports, Science and Technology
as part of a progress update report of Grant No. 23531201
for Scientific Research (C) (General) (2011-2014) entitled
Developing a Core Curriculum for the Dental English Course in all dental schools in Japan
日本の大学歯学部（歯科大学）での歯科英語教育におけるコアカリキュラムの開発
「基盤研究（C）（一般）（H23～H25）」


Principal Investigator:

**Omar Marianito Maningo Rodis**

Institute of Health Biosciences
The University of Tokushima Graduate School

## CO-INVESTIGATORS

| | | | |
|---|---|---|---|
| 1. | **Okayama University Hospital**<br>岡山大学病院 | **Assistant Professor**<br>助教 | **Michiko Nishimura**<br>西村 美智子 |
| 2. | **Okayama University Hospital**<br>岡山大学病院 | **Assistant Professor**<br>助教 | **Naoyuki Kariya**<br>假谷 直之 |
| 3. | **Okayama University**<br>岡山大学医療教育統合開発センター | **Assistant Professor**<br>助教 | **Toshiko Yoshida**<br>吉田 登志子 |

## COLLABORATORS

| | | | |
|---|---|---|---|
| 1. | **Health Sciences University of Hokkaido**<br>北海道医療大学 | **Professor**<br>教授 | **Yujiro Handa**<br>半田 祐二朗 |
| 2. | **Hokkaido University**<br>北海道大学 | **Assistant Professor**<br>助教 | **Taro Arima**<br>有馬太郎 |
| 3. | **Hokkaido University**<br>北海道大学 | **Associate Professor**<br>准教授 | **Shigeru Takahashi**<br>高橋 茂 |
| 4. | **Iwate Medical University**<br>岩手医科大学 | **Professor**<br>教授 | **Yoshinori Sahara**<br>佐原 資謹 |
| 5. | **Iwate Medical University**<br>岩手医科大学共通教育センター | **Associate Professor**<br>准教授 | **James Hobbs**<br>ホッブスジェイムズ |
| 6. | **Tohoku University**<br>東北大学 | **Assistant Professor**<br>助教 | **Yoshinaka Shimizu**<br>清水 良央 |
| 7. | **Ohu University**<br>奥羽大学 | **Professor**<br>教授 | **Naomi Fukai**<br>深井 直実 |
| 8. | **Nihon University at Matsudo**<br>日本大学松戸 | **Associate Professor**<br>准教授 | **Tomiko Yamagami**<br>山上 登美子 |
| 9. | **Tokyo Medical and Dental University**<br>東京医科歯科大学 | **Assistant Professor**<br>助教 | **Naoko Seki**<br>關 奈央子 |
| 10. | **Tokyo Dental College**<br>東京歯科大学 英語研究室 | **Associate Professor**<br>准教授 | **Yoshiaki Shibaie**<br>柴家 嘉明 |
| 11. | **Nippon Dental University – Tokyo**<br>日本歯科大学東京 | **Associate Professor**<br>准教授 | **Chie Yanai**<br>柳井 智恵 |

| | | | |
|---|---|---|---|
| 12. | **Nippon Dental University – Tokyo**<br>日本歯科大学東京 | **Intern**<br>研修医 | **Michael Ishii**<br>石井 マイケル |
| 13. | **Nihon University School of Dentistry**<br>日本大学歯学部 | **Professor**<br>教授 | **Clive Langham**<br>ラングハムクライブ |
| 14. | **Showa University**<br>昭和大学富士吉田校舎 | **Lecturer**<br>講師 | **Masaki Ohno**<br>大野真機 |
| 15. | **Showa University Dental School**<br>昭和大学歯学部 | **Assistant Professor**<br>助教 | **Yo Shibata**<br>柴田 陽 |
| 16. | **Kanagawa Dental University**<br>神奈川歯科大学 | **Professor**<br>教授 | **Martin Peters**<br>ピータースマーティン |
| 17. | **Tsurumi University**<br>鶴見大学 | **Assistant Professor**<br>助教 | **Asiri Jayawardena**<br>ジャヤワルディナアスリ |
| 18. | **Niigata University**<br>新潟大学 | **Associate Professor**<br>准教授 | **Roxana Stegaroiu**<br>ステガロユロクサーナ |
| 19. | **Nippon Dental University – Niigata**<br>日本歯科大学新潟生命歯学部 | **Professor**<br>教授 | **Ikuo Kageyama**<br>影山 幾男 |
| 20. | **Asahi University**<br>朝日大学 | **Professor**<br>教授 | **Hironori Tsuchiya**<br>土屋 博紀 |
| 21. | **Aichi Gakuin University**<br>愛知学院大学 | **Lecturer**<br>講師 | **Kazuyoshi Suzuki**<br>鈴木 一吉 |
| 22. | **Osaka Dental University**<br>大阪歯科大学 | **Associate Professor**<br>准教授 | **Junichi Fujita**<br>藤田 淳一 |
| 23. | **Okayama University**<br>岡山大学 | **Associate Professor**<br>准教授 | **Seishi Matsumura**<br>松村誠士 |
| 24. | **Hiroshima University**<br>広島大学 | **Specially-appointed Assistant Professor** 助教 | **Hiroko Oka**<br>岡 広子 |
| 25. | **Kyushu University**<br>九州大学 | **Lecturer**<br>講師 | **Jane Harland**<br>ハーランドジェーン |
| 26. | **Nagasaki University**<br>長崎大学 | **Assistant Professor**<br>助教 | **Etsuko Watanabe**<br>渡邊 悦子 |

# ADVISERS

## J. Patrick Barron　バロン・パトリック

Professor and Chair, Department of International Medical Communications, Tokyo Medical University
Secretary General, World Association for Bronchology
Editorial Consultant, Journal of Gastroenterology
Vice-President, World Bronchology Federation
Consultant, International College of Surgeons (Japan Chapter)
Strategic Planning Committee, Asian Pacific Society for Respirology
Editorial Board, CHEST
Director, Japanese Society of Travel Medicine
Director, Kenko to Onsen Forum
Advisory Panel for Medical Communications, Seoul National University Bundang Hospital
Editorial Board, Respirology
Editorial Board, The Japanese Journal of Gastroenterological Surgery

## Edward Barroga　バロガ・エドワード

Associate Professor and Senior Editor, Department of International Medical Communications, Tokyo Medical University
PhD, Hokkaido University Graduate School of Veterinary Medicine
DVM, University of the Philippines
Assistant Professor and Head, Pathology Laboratory, University of the Philippines
Chairman and Associate Professor, Department of Veterinary Para-clinical Sciences, UP
Biomedical Editor, MYU Research
Senior Editor, ThinkSCIENCE, Inc.

## Chieri Noda　野田 千ゑ里

Senior Lecturer, Department of International Medical Communications, Tokyo Medical University
MA, Birkbeck, University of London
Communicative strategies used by medical researchers in the UK and Japan

## Aya Watanabe　渡邉　綾

Research Associate, Department of International Medical Communications, Tokyo Medical University
Bilingualism, Second Language Acquisition and English Education, Department of English Language and Studies at Sophia University
MA,  University of Hawaii at Manoa
Conversation Analysis as a methodology to investigate classroom interaction and language acquisition

**PREFACE**

In April 2007, the Japan Society for the Promotion of Science (JSPS) Project Team for Supporting University Internationalization released their interim report on Innovative Models for Promoting the Internationalization of Japanese Universities. The message by the JSPS President, Prof. Motoyuki Ono, upon his meetings with foreign university and government officials stated that “in all countries, the issue of university internationalization is recognized as a matter of some urgency, and that this recognition is accompanied by active engagement in the cause of internationalization by both the universities themselves and government officials”. The interim report’s conclusion stated that “According to the International Association of Universities’ (IAU) survey of universities around the world, lack of faculty interest and involvement was often cited as an “impediment to university internationalization”. This suggests that building “internal consensus” is the most important issue for university internationalization, particularly for “internal internationalization”. It further stated that “JSPS hopes to use symposiums and other forums to introduce Japanese universities’ internationalization efforts to a wider audience, share information and provide opportunities for exchange of opinions”.

This will be the first core curriculum on dental English courses, prepared by educators from different dental schools in Japan, to be used as a guide for English education in dentistry. The teachers participated in the first ever fact-finding discussion forum addressing the problems facing dental English education and building internal consensus as to what the course comprises. Its membership includes native and non-native English teachers, dentists, lecturers and professors from dental schools in Japan. The core will be endorsed to the Ministry of Education, Culture, Sports, Science and Technology (MEXT) during the 3rd meeting. The 1$^{st}$ meeting was held at Okayama International Center in Okayama on August 28, 2011, as a discussion forum and was the first ever meeting of educators interested in improving English education in dentistry. The 2$^{nd}$ meeting was held at Tokyo Medical and Dental University in Tokyo on June 9, 2012. Guest speakers from the Department of International Medical Communications, Tokyo Medical University were invited and acted as advisers during the workshop.

It is recommended that this guide should only be used as basis for developing and improving syllabus design specific and appropriate to each dental school. The needs of schools, students and teachers are continuous and it is therefore inevitable for schools and education officials to regularly review and assess each curriculum based on teaching and learning experiences of teachers and students.

The views or opinions contained within this report are those of the participants and experts convened for the discussion forum and workshop and are not necessarily the official views or opinions of their respective schools.

## ACKNOWLEDGMENTS

We wish to thank the heads and personnel of the Academic Affairs Section of the 29 dental schools in Japan.

We would also like to thank the following distinguished professors and academicians who shared information about dental education in their respective universities/countries: Kotsanos Nikolaos, Aristotle University (Greece); Daniel Reißmann, University Medical Center Hamburg-Eppendorf (Germany); Daisylin Konle, Marlis Walther and J. Thomas Lambrecht, University of Basel/Bern (Switzerland); Juha Varrela, University of Turku (Finland); Katalin Gabor, University of Debrecen (Hungary); Rodivick Docor, Southwestern University (Philippines); Zac Morse, University of Hong Kong (China); Lihong Ge, Peking University (China); Ying Ji, Dalian University (China); Zann Lum, National University of Singapore (Singapore); Helen Ngu, University Sains Malaysia (Malaysia); Kazi Anisur Rahman, Bangladesh Dental College (Bangladesh); Asiri Jayawardena, Peradeniya University (Sri Lanka); Chitta Ranjan Choudhury, Nitte University AB Shetty Memorial Institute of Dental Sciences (India), Shun-Te Huang, Kaohsiung Medical University (Taiwan); Baek-il Kim, Yonsei University (Korea); Ulamnemekh Hulan, Health Science University of Mongolia (Mongolia); Jinda Lertsirivorakul, Khon Kaen University (Thailand); Wael Amgad Hassan, Misr International University (Egypt); and Tarek Balam, Benghazi University (Libya) / Damascus University (Syria).

## TABLE OF CONTENTS


**UNIT 1 INTRODUCTION**

1.1 Background .......... 1
1.2 Rationale for Developing the Core Curriculum .......... 2
1.3 Objectives for Developing the Core Curriculum .......... 7

**UNIT 2 THE CORE CURRICULUM**

2.1 Curriculum Framework .......... 9
2.2 Module 1: Basic Terminology and Conversation .......... 10
2.2.1 General Instructional Objective (GIO) .......... 10
2.2.2 Specific Behavioral Objectives (SBOs) .......... 10
2.2.3 Medical and Dental Terminology (Basic) .......... 10
2.2.4 Introduction to Dentistry and Oral Anatomy .......... 11
2.2.5 Peer Interaction (Beginners) .......... 12
2.3 Module 2: Advanced Terminology and Conversation .......... 13
2.3.1 General Instructional Objective (GIO) .......... 14
2.3.2 Specific Behavioral Objectives (SBOs) .......... 14
2.3.3 Medical and Dental Terminology (Advanced) .......... 14
2.3.4 Oral Anatomy and Function (Advanced) .......... 15
2.3.5 English Conversation in Dentistry (Academic/Clinical) .......... 16
2.3.6 Computer-based Learning (E-Learning) .......... 17
2.3.7 Peer Interaction (Advanced) .......... 19
2.4 Learning Strategies (LS) .......... 20
2.5 Evaluation (EV) .......... 20

**UNIT 3 SAMPLE SYLLABUS**

3.1 Module 1
3.1.1 One-Semester course .......... 21
3.1.2 Two-Semester course .......... 22
3.2 Module 2
3.2.1 One-Semester course .......... 24
3.2.2 Two-Semester course .......... 25
3.3 Sample Syllabus with LS and EV .......... 27

**UNIT 4 IMPLEMENTATION** .......... 29

**UNIT 5 EVALUATION** .......... 30

**UNIT 6 REFERENCES** .......... 31

**UNIT 7 APPENDICES** .......... 33

- Survey for Dental Schools
- Current Status and Distribution of Dental English Education
- The 1st and 2nd Meeting Brochure and Program
- The 1st and 2nd Meeting Interim Reports

# UNIT 1: INTRODUCTION

## 1.1 Background[1]

Globalization and demographic changes in Japan have created a situation in which future professionals have begun to foresee themselves as part of international scientific exchange and communication. In 1989, a curriculum guideline called "Course of Study" was developed by Japan's Ministry of Education, Culture, Sports, Science and Technology (MEXT).[2] The guideline called for a shift from mastering grammar toward an emphasis on functional, communication-oriented teaching and the development of a student's listening and speaking skills. This guideline was revised in 1999. Then, in 2003, MEXT presented a national guideline, which aimed to improve the quality of English education in Japan and produce citizens who can function effectively and be competitive in global society.[2] This led to higher education reforms among universities aiming to produce unique and marketable education programs locally and internationally. English became integrated into the curricula of different professional fields. This led further to the establishment of English for Specific Purposes (ESP), English for Occupational Purposes (EOP), and English for Academic Purposes (EAP) courses in medical, paramedical, and technical fields in Japan.

Dental English courses were eventually introduced into Japanese dental curricula to address the increasing demand for international scientific exchange and communication. Such courses are variously known in dental schools as Dental English, English for Dentistry, English for Dental Medicine, *Eigo Shigakubu, Shigaku Eigo*, and *Shika Eigo*. Since such courses lack standardized nomenclature, they will be referred to as dental English courses in this report. These courses aim to teach English dental terminology and present conversational situations commonly used in the field of dentistry. Unfortunately, not all of Japan's 29 dental schools offer such courses. For schools that offer these courses, there is a disparity as to what it comprises, when it is offered, and how it is taught. Additionally, a core curriculum, which specifically prescribes the content of these courses, has yet to be developed. This report presents a possible basis for a common core curriculum for dental English courses taught in Japan by highlighting the current situation and needs among dental

schools and students. This report also summarizes the feedback of participants representing their respective dental schools during the 1st discussion forum held on August 28, 2011.

## 1.2 Rationale for Developing the Core Curriculum

Dental education in Japan is supervised and accredited by MEXT; it consists of a six-year program consisting of didactic and practical courses, including two years of pre-dentistry and four years of dentistry subjects.[4] Depending on the university, candidates applying to a dental school would have had spent at least six years studying English in high school and junior high school, pass at least one entrance examination with English as a compulsory or elective subject, and will have one or two more years learning English in the university. Komabayashi et al. presented a sample dental curriculum of a private dental school in Japan showing the respective curriculum hours per subject.[5] Upon attaining their sixth year in dental school, students become eligible to take the national dental licensure examinations, which are given out in Japanese.

To assess the current situation of English education in dental schools, we conducted a survey in March of 2011. The survey was an eight-item bilingual (English and Japanese) questionnaire addressed to the head of the academic affairs at each of Japan's 29 dental schools (see Appendix). An introductory letter explaining the purpose of the survey was attached to the questionnaire. The eight questions were part of a comprehensive survey on English language education in Japanese dental schools conducted by Morse and Nakahara in 1999.[6] However, the said survey did not differentiate between general English and dental English courses taught within the dental curriculum. In our current survey, instructions were clearly stated to differentiate between general English and dental English courses. The eight questions were as follows: (1) Do you offer a dental English course? (2) When is it offered? (3) How many minutes does a class last? (4) What is the average number of students per class? (5) How many dental English educators do you have? (6) What is the professional background of educators? (7) Do you use official textbooks? And (8) Do you offer elective dental English classes to postgraduate students?

A 100% response rate was achieved within one month (29/29 dental schools). Table 1 shows a list of the 29 dental schools in Japan: the 1st to 12th listed items are public schools; the 13th to 29th are private schools; the schools are listed geographically within each category. As of March 2011, 22 schools offered a dental English course, of which nine were public schools and 13 were private.

**Table 1** **The current status of dental English courses taught in Japan's 29 dental schools** (as of March 2011)

| | School | Offer Course | When Offered (Year $^{Semester}$) | Class Hours (minutes) | Ave. # of Students | # of Teachers | Background of Teacher | Offer to Postgrads | Textbook |
|---|---|---|---|---|---|---|---|---|---|
| 1 | Hokkaido University | no | x | x | x | x | x | yes | x |
| 2 | Tohoku University | yes | $5^{1}$ | 50 | 55 | 11 | others | yes | x |
| 3 | Tokyo Medical and Dental University | yes | $1^{2}$ $2^{12}$ $3^{1}$ $4^{1}$ $5^{1}$ | 90/50 | 55 | 4 | linguist/dentist | no | None |
| 4 | Niigata University | yes | $3^{12}$ $4^{2}$ | 90 | 40 | 1 | dentist | yes | Dental Chair-side Communication, etc. |
| 5 | Osaka University | no | x | x | x | x | x | yes | x |
| 6 | Okayama University | yes | $1^{2}$ | 90 | 52 | 2 | dentists | no | None |
| 7 | Hiroshima University | yes | $3^{12}$ $4^{2}$ | 90 | 55 | 16 | dentists/others | yes | None |
| 8 | Tokushima University | yes | $3^{2}$ | 60 | 45 | 20 | dentist | no | Language of Medicine |
| 9 | Kyushu Dental College | yes | $3^{2}$ $5^{1}$ | 90 | 87 | 2 | others | no | Effective Academic Writing |
| 10 | Kyushu University | yes | $1^{12}$ $2^{2}$ $3^{12}$ $4^{12}$ | 90 | 60 | 2 | linguist/dentist | yes | Kyushu University Dental English Series |
| 11 | Nagasaki University | yes | $1^{2}$ | 180 | 50 | 2 | dentists | no | None |
| 12 | Kagoshima University | no | x | x | x | x | x | no | x |
| 13 | Health Sciences University of Hokkaido | yes | $2^{2}$ $3^{1}$ $5^{1}$ | 80 | 60 | 1 | health | no | Concise Human Body |
| 14 | Iwate Medical University | yes | $2^{12}$ $3^{1}$ $4^{12}$ | 90 | 80 | 15 | ling/dent/others | yes | A Way to Good Health |
| 15 | Ohu University | yes | $1^{12}$ $2^{12}$ | 60 | 100 | 2 | linguist/others | yes | None |
| 16 | Meikai University | yes | $1^{12}$ $2^{12}$ $3^{1}$ $4^{1}$ $6^{12}$ | 90 | 120 | not stated | dentist/others | no | Yes (not stated) |
| 17 | Nihon University Matsudo | no | x | x | x | x | x | yes | x |
| 18 | Tokyo Dental College | yes | $2^{1}$ $3^{1}$ | 90 | 98 | 1 / 14 | linguist/dentist | no | Yes (not stated) |
| 19 | Nippon Dental University Tokyo | yes | $2^{12}$ | 80 | 147 | 2 | others | no | Yes (not stated) |
| 20 | Nihon University School of Dentistry | yes | $2^{12}$ $3^{1}$ | 50 | 80 | 2 / 6 | linguist/dentist | no | None |
| 21 | Showa University | yes | $2^{12}$ $3^{1}$ | 90 | 30 | 7 | others | yes | Speaking of Speech |
| 22 | Tsurumi University | yes | $2^{12}$ | 85 | 38 | 4 | dentist/others | yes | Oxford English for Career Med |
| 23 | Kanagawa Dental College | yes | $2^{2}$ $3^{1}$ | 75 | 95 | 1 | others | yes | None |
| 24 | Nippon Dental University - Niigata | yes | $1^{12}$ $2^{12}$ | 80 | 70 | 2 | linguist | no | Understanding Dentistry |
| 25 | Matsumoto Dental University | yes | $1^{12}$ $2^{12}$ | 90 | 50 | 1 | others | yes | Access to Simple English |
| 26 | Asahi University | yes | $1^{12}$ | 90 | 75 | 1 | others | no | Yes (not stated) |
| 27 | Aichi Gakuin University | no | x | x | x | x | x | no | x |
| 28 | Osaka Dental University | no | x | x | x | x | x | no | x |
| 29 | Fukuoka Dental College | no | x | x | x | x | x | no | x |

Table 2 indicates the year level and semester the courses are offered among those schools that provide them. Most of the public schools offered a dental English course in the second semester of the first and third year and the first semester of the third and fifth year; most private schools offered a course until the first semester of the third year, though some offered one in the fourth to sixth years of dental school.

**Table 2.** **Current distribution of dental English education per year level** (For the 22 schools offering the course)

| | 1st year | | 2nd year | | 3rd year | | 4th year | | 5th year | | 6th year | |
|---|---|---|---|---|---|---|---|---|---|---|---|---|
| | 1st sem | 2nd sem | 1st sem | 2nd sem | 1st sem | 2nd sem | 1st sem | 2nd sem | 1st sem | 2nd sem | 1st sem | 2nd sem |
| **Public** (25 course slots) | ● | ●●●● | ● | ●● | ●●●● | ●●●●● | ●● | ●●● | ●●● | | | |
| **Private** (44 course slots) | ○○○○○ | ○○○○○ | ○○○○○<br>○○○○○ | ○○○○○<br>○○○○○<br>○ | ○○○○○<br>○○ | | ○○ | ○ | ○ | | ○ | ○ |
| **All** (69 course slots) | ●○○○○<br>○ | ●●●●○<br>○○○○ | ●○○○○<br>○○○○○<br>○ | ●●○○○<br>○○○○○<br>○○○ | ●●●●○<br>○○○○○<br>○ | ●●●●● | ●●○○ | ●●●○ | ●●●○ | | ○ | ○ |

● Public schools
○ Private schools

Half of the schools held courses in the form of 90-minute classes; the range with the remainder was 50–180 minutes. The number of students per class was observed to be lower in public schools (mean=50) than in private schools (mean=80). The number of instructors teaching the course varied from one teacher in charge of three or four semesters to as many as 20 in charge of one semester. The professional background of the instructors also varied: some schools had dentists, whereas other schools have language specialists, teachers with different backgrounds, or a mixture of all three. Of the 22 schools, 14 offered a course that used textbooks, but very few employed one that was specifically written for dental students; this confirms the results reported in the Morse and Nakahara study[6]. Some books exist that deal with conversations commonly used in dentistry, but most are for advanced students or those who have already studied dental terminology. Almost half of the 29 schools offered dental English as a postgraduate elective course to their students, including some schools that did not offer such a course for undergraduates.

An article by McVeigh identified some problems that prevent higher education policy improvements in Japan, such as academic accountability, student opinion, and bureaucracy.[7] This observation was backed by a recent article by Stapleton — "Japanese Universities: Change or risk marginalization"— in which the author pointed out that these problems contribute toward the prevailing mediocrity and failure to take the fullest advantage of the talent (current needs of students) that exists in Japanese universities.[8] Stapleton's paper adds that with regard to higher education among developed countries, Japan is an outlier. Some medical and dental schools in Asian countries have long operated an all-English curriculum for their students, and most dental schools in the Middle East have their curricula entirely in English. In many dental schools in Hong Kong, Singapore, Bangladesh, India, Malaysia, Sri Lanka and the Philippines, dental subjects are entirely taught in English, and the national board examinations are given out in English as well. Among other Asian countries, there are also schools that teach dentistry in the national language but use English textbooks as references and include English questions in their board examinations. For instance, at Kaohsiung Medical University in Taiwan, the language of instruction is Mandarin, but English textbooks are used, and the national board examinations include both

Mandarin and English terminology. In Korea, China, Mongolia, and Thailand, instruction is conducted entirely in the national language, though a growing number of dental schools in these countries have started to increase course slots for English for specific or academic purposes in their curricula. At Yonsei University in Korea, first-year medical and dental students are required to take their liberal arts subjects in English at a special residential campus for one year with a foreign student as a roommate. The university terms this learning process “English Immersion Education.” The curriculum for the second year onward is under review by officials at this university toward the inclusion of more specific dental English courses. In China and Mongolia, although all subjects are taught in the national language, plans to increase credits for scientific English have started. At Khon Kaen University in Thailand, all subjects, including the board examinations, are in Thai. However, five English courses have been included in its current dental curriculum: English for Health Sciences I (three credits in the first semester of the first year); English for Health Sciences II (three credits in the second semester of the first year); Technical English for Dentistry (two credits in the second semester of the second year); Writing English for Health Sciences (two credits in the second semester of the fifth year); and Speaking English for Health Sciences (two credits in the first semester of the sixth year). One credit is equal to one hour per week for 15 weeks. These subjects are offered in the first, second, fifth, and sixth years of dental school.

In 2001, the European Union’s statistical unit Eurostat found that more than 90% of pupils in secondary schools in the EU study English, which is believed to occupy a vital role in Europe’s basic education systems from primary education onwards. Because of this, students in countries like Switzerland, Finland, Germany and Greece, where the medium of instruction of the dental curriculum is in the national language, may have fewer difficulties in mastering English dental terms. They also study Latin as part of the dental curriculum, which helps them with Latin-derived technical terms in English. Thus, at the universities of Bern and Basel in Switzerland, the University of Turku in Finland, the University Medical Center Hamburg-Eppendorf and Ludwig-Maximilian University in Germany and Aristotle University in Greece, the dental English course is offered only as an elective subject in the curriculum. However, other

schools in Europe may have had established compulsory dental English courses in their respective curricula. The University of Debrecen Medical and Health Science Center in Hungary for instance, offers 120-class-course in dental English.

An English-medium university is the ideal place for reforms in both higher education and English education if competency and globalization are to be achieved. At such a university, all minor and major courses have English as the medium of instruction.[9] However, for medical and dental schools in Japan, this poses a problem since clinical practical examinations and the national board examinations are conducted in Japanese. In contrast, dental curriculum in the Philippines, Singapore, and India is taught entirely in English, and the national dental board examinations are also given in English. In actual clinical practice however, dentists from these countries communicate with their patients in the local language. Nonetheless, when these dentists pursue practice or study abroad, present at international conferences, or read/write scientific papers for continuing education purposes, they usually find it less of an obstacle to catch up with their international counterparts than do Japanese dentists.

In April 2010, the Science Council of Japan published a report on the necessity of English education in the dental field as a response to globalization.[10] The report stated that since English is the international language, English proficiency will be necessary to enhance research and education in Japan. Most researchers from Japan have always felt some anxiety about presenting their results at international conferences owing to a lack of confidence in their English-speaking skills, and most prefer to do poster presentations.[11] Recently however, poster presenters at scientific conferences are now required to provide a brief oral presentation of their studies followed by the question-and-answer session. International conferences the world over clearly state that English is the official conference language so dental schools where English is not the official language of instruction have begun incorporating dental English courses into their curricula to meet this state of affairs. English education can also provide opportunities to Japanese researchers as evidenced by the fact that half of Japan’s Nobel Prize winners made a career in the United States.

Ideally, students should identify their future needs—whether for treating foreign patients, academic or research career development, or inter-professional communication. The dental professional field in Japan as a whole therefore needs to be reassessed, taking into consideration the current needs of students, schools, teachers, dentists, society (both local and foreign patients), education officials, and the nature of dental English courses.[12,13] If the various challenges are addressed, future Japanese dentists will be able to conduct professional and patient communication at an international level.

It is therefore the recommendation of this grant that a gathering of school officials from each of the 29 dental schools be organized to facilitate a fact-finding and discussion forum concerning the need to develop a core curriculum for dental English education. Since most schools regularly assess their student's needs as well as the necessity for globalization of the profession, this forum will pave the way toward drafting a suitable outline of the curriculum. The agenda should include the setting of general and specific instructional objectives, the structure and content of the curriculum, and the sharing of teaching and learning methods by teachers of the courses. It should also include basic and technical vocabulary, reading, writing, speaking and other confidence-building activities to motivate students. Since the Morse and Nakahara study in 1999[6], dental English courses have continued to be an area of dentistry in Japan that has received little attention. Up to now, such courses still lack the basis of a recommended core curriculum, leaving syllabus design to individual schools or teachers. If the aim of MEXT in producing professionals who can function effectively and be competitive in the global society is to be met, there should be regular and systematic fact-finding discussion forums by school and education officials to assess and improve courses that need attention and then implement them.

### 1.3 Objectives for Developing the Core Curriculum

The main objective in developing a core curriculum for dental English courses is to provide teachers and education officials with an officially approved curriculum to base on, or improve further, appropriate to their needs, that of the school, and their

students. The core should address the ever-changing needs and concerns of the profession and the patients. It should therefore have courses that will develop vocabulary, inter-profession and patient-dentist communication and student-motivation. This will be made possible through a gathering of teachers of Dental English courses from Japan's 29 dental schools to discuss how to develop and implement a core curriculum for the course based on their own experiences, a consensus on what should comprise the course, the establishment of the current needs of the respective schools, and the needs of their students. In addressing the need for teachers to be continually updated with the ever changing needs of the course, an additional objective to organize a support group for teachers is envisaged.

## UNIT 2: THE CORE CURRICULUM

### 2.1 Curriculum Framework

The proposed core curriculum will address the challenges of globalization of the profession. It is divided into two modules of didactic and practical courses offered in any of the pre-dental years (from 1st to 2nd year) and in any of the dental years (from 3rd to 6th year) of the 6-year dental curriculum. It will cover basic and advanced terminology and conversation aimed at improving self-confidence, dentist-patient and inter-professional communication. Courses in general and dental terminology will include etymology (Greek/Latin derivatives) and principles of terminology (prefix/suffix, combining vowels, and using terms in context). Each module can be offered as a one-semester or two-semester course. Thus, each school can modify the proposed core curriculum according to their needs and resources, as long as the same objectives are used.

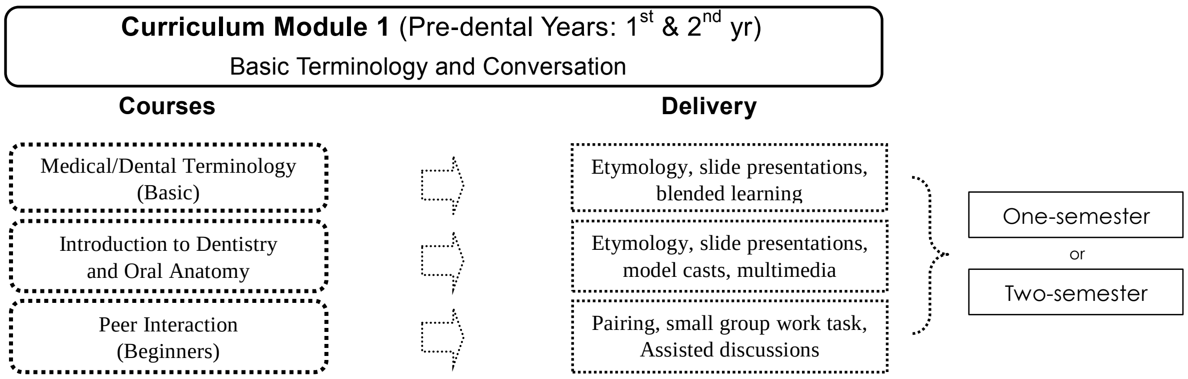


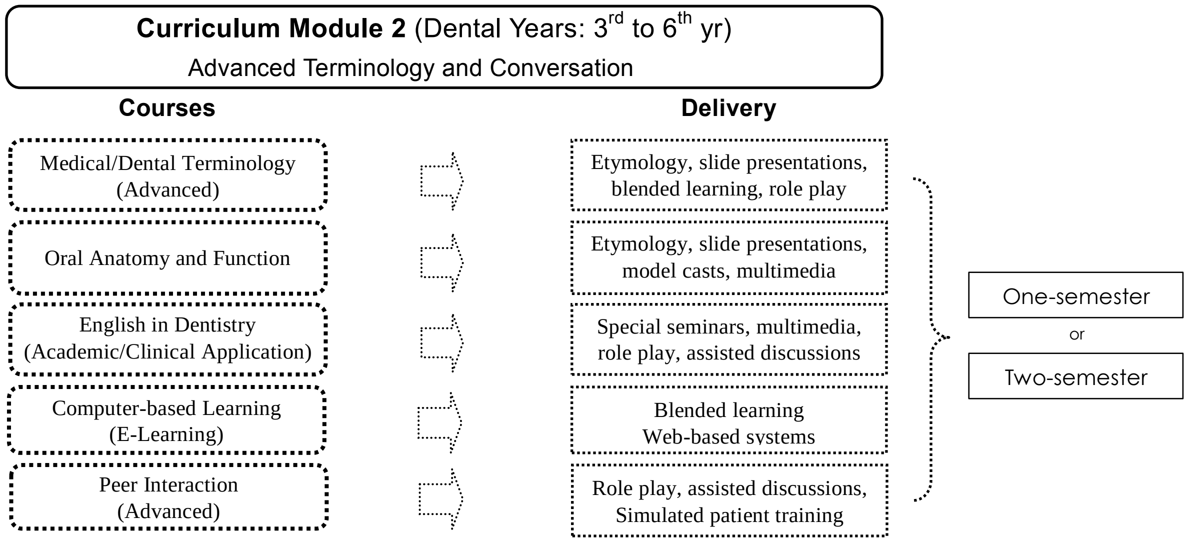

## 2.2 **Curriculum Module 1:** Basic Terminology and Conversation

Module 1 may be offered in any of the semesters during the pre-dental years, which is from the 1st year to the 2nd year. This first 2 years of the 6-year dental curriculum mainly cover the basic sciences and a few orientation courses on dentistry. Module 1 contains courses covering common words used in the daily interaction with patients, introduction to dentistry, basic medico-dental terms and their etymology, and simple interactive exercises to motivate them of the importance of professional globalization.

### 2.2.1 General Instructional Objective (GIO)

Students will learn how to communicate with English-speaking patients on basic topics of routine dental consultation and treatment.

### 2.2.2 Specific Behavioral Objectives (SBOs)

1. Students will be able to say and respond to basic dental terms and phrases.
2. Students will be able to understand what the patient says in English.
3. Students will be able to ask their patients about their medical and dental conditions as well as explain dental procedures in English.
4. Students will be able to access and use fact sheets for native speakers at websites, for example, international dental associations, international journals, etc.

### 2.2.3 Medical and Dental Terminology (Basic)

*Course description*

The course will cover basic medical and dental terms commonly used in general situations of daily life. Having the course right after the students graduate from high school will complement or prepare them in improving their English skills in the health sciences. It will include casual and formal ways of greeting, speaking and listening. Additionally, knowledge of common general terms such as dental clinic, reception, appointment as well as medical terms such as fever, cold, diabetes, etc. is included as it is an integral part of history-

taking, diagnosis, treatment and patient-dentist or inter-professional interaction. Significant part of the course will emphasize on the etymology of commonly used terms with a focus on overall meaning.

*Delivery*

Teaching methods include blended learning, presentations and etymology. Blended learning emphasizes learning through the use of technology and face-to-face interaction. Although gaining popularity, there are considerations that have to be met in blended learning. This approach emphasizes learning through the use of technology such as access to online resources, communication via social media or interaction with distance learners in other classrooms, use of audio-video files, and face-to-face interaction. Although gaining popularity, there are considerations that have to be met in blended learning. This includes the resources available, the competency and nature or location of the students. Slideshow presentations should be customized to the level of understanding of the students. This will be an effective tool in enhancing better understanding and memory retention of new topics. Subtitles on slides with new or technical terminology should be used to augment student comprehension as visual aids. The medium of instruction should be English followed by a very brief translation in Japanese to emphasize the conveyed message particularly for new terms. English must be pronounced clearly and slowly. For technical terms, it is necessary for students to learn the basic principles in terminology structure since medical/dental terminology include Latin or Greek root words, prefixes, suffixes and combining vowels.

### 2.2.4 Introduction to Dentistry and Oral Anatomy

*Course description*

The course focuses on the the history, scope and the science of dentistry and the basic nomenclature of the teeth and its supporting structures. Topics will include the introduction of dentistry as a science and profession plus the names of the types of teeth and the structures surrounding it.

*Delivery*

Teaching methods include word etymology, slideshow presentations and use of model casts or multimedia. Etymology is important in understanding the meanings of terms commonly used in medicine and dentistry. The term "oral" and "cavity", for example, are both derived from the Latin word "*oralis*" and "*cavus*", respectively. "*Oralis*" means mouth while "*cavus*" means hollow. Understanding the meaning of the word roots can give students an idea of what the final term would mean. In this case, "oral cavity" would mean the mouth, which is hollow. In addition, any word with "*oralis*" (as in Streptococcus oralis) or "oral" (as in oral anatomy) can tell the learner that it pertains to the mouth while any word with "*cavus*" (as in cavus foot or highly arched foot) and "cavity" (as in dental cavity) pertains to something hollow. Slideshows should contain more pictures or videos of the teeth and its related structures to enhance learning and interest of students. The use of model casts is also one way of enhancing the learner's cognitive learning through visual input.

2.2.5 Peer Interaction (Beginners)

*Course description*

The course focuses on pairing or grouping students to perform specific tasks or roles for a given simple dentist-patient situation. It has been proven that effective interaction with peers becomes a successful and powerful learning method only if students encourage each other to ask or answer questions, explain and express their thoughts. However, the majority of Japanese students feel uneasy about expressing their thoughts in public, much more in English. The beginners course must therefore introduce to students the importance of improving one's social skills, which they will need in their future professional interactions, through assisted discussions, peer pairing or small group-work tasks. Activities should include situations in casual and formal greetings, group discussions on simple topics of interest, inquisitiveness exercises, gesturing, facial expressions and culture studies, among others.

*Delivery*

Almost all conversations, whether they be formal or informal, start with a greeting. The same is applicable when meeting patients or colleagues for the first time, seeing the patients again for recall, self introduction, and introducing others. Expressions of farewell and well-being are equally important. Role playing by pairs consists of situations like meeting someone for the first time, meeting someone again, or introducing your friend to others. Role playing by small groups can be about trying to know someone in class you do not know and introducing him/her to the class. Most of the students may already know each other but it is important to emaphasize to them that the purpose of the exercise is to practice their English listening and speaking skills.

Assisted discussions may be performed by reading and discussing simple cases in daily living among the group with teacher assistance. Inquisitieveness exercises include having students go around and ask questions to each other or having them give out their self-formulated quizzes to their classmates. This exercise will prepare them in gaining confidence in asking questions in future classes. Lectures and practical exercises on gesturing, facial expressions and culture studies should also be included since these also form an important part of conversation.

## 2.3 **Curriculum Module 2:** Advanced Terminology and Conversation

Module 2 may be offered in any of the semesters during the dental years, which are from the 3rd year to the 6th year. The last 4 years of the 6-year dental curriculum mainly cover didactic and clinical dentistry. Module 2 contains courses covering terms used in the patient- and inter- and intra-profession interaction, etymology of advanced medical and dental terms, and interactive exercises focusing on dental clinical, academic, research and international situations. This will provide students with the basic knowledge needed for self-confidence, self-improvement, dentist-patient communication and inter-professional communication.

Self-confidence – having adequate knowledge of terms

Self-improvement – be competent in acquiring and sharing new knowledge
Dentist-patient communication – build strong communication skills
Inter/intra-professional communication – interact with international colleagues

2.3.1 General Instructional Objective (GIO)

Students will acquire the basic skills of professional communication

2.3.2 Specific Behavioral Objectives (SBOs)

1. Students will be able to say and respond to technical (dental) terms and phrases.
2. Students will be able to create posters on dental topics.
3. Students will be able to make oral presentations on dental topics.
4. Students will be able to correspond with other health professionals. (For example: e-mail, lectures, meetings, etc.)

2.3.3 Medical and Dental Terminology (Advanced)

*Course description*

The course will cover more advanced/technical word lists of general, medical and dental terminology and studying in context these terms and sentence structures from different conversational situations. Since most medical terminology and dental terminology are derivatives of the Greek or Latin languages, it is imperative to study etymology, root words, prefix, suffix and combining vowel terminology structure.

*Delivery*

Teaching methods will still include blended learning and etymology. As with Module 1, teaching new terms should focus on overall meaning and not on memorization so if the situation or flow of conversation changes, students will still be able to understand or rephrase their statements accordingly. This is the reason why teaching terminology should be taught in context, and not just the term per se. Teaching should be supplemented by multimedia (video, audio, slideshow presentations) and if possible, pronunciation lessons and exercises.

For example, the term “Oral Pathology”:

Oral (from Latin *oralis*) + Patho (from Greek *pathos*) + logy (from Greek *logia*)

↓ ↓ ↓

| | | | |
|---|---|---|---|
| **English meaning**: | mouth | disease | study of |
| **Japanese meaning**: | 口腔 | 病気 | ～の（に関する）学問（科学） |
| **Dental term**: | **oral** | **patho** | **logy** |
| **Pronunciation**: | **awr**-*uh*l | p*uh*-**tho** | **l**-*uh*-jee |

- knowledge of the etymology of most medical and dental terms will allow students to better understand the principles of terminology than rote learning or memorization. Additionally, teaching terms in context will help students understand the use of the term better. In the example above, students will know that any term with “oral” (ex. oral health) pertains to health of the <u>mouth</u>, any term with “patho” (ex. pathologic conditions) pertains to the study of <u>disease</u> conditions, and any term with “logy” (ex. biology) pertains to the <u>study of</u> life [*bio* = life]. Teachers can make use of blended learning to change/substitute the terms or meanings so students can improve comprehension. Pronunciation guides or audio clips may be inserted into slideshow presentations together with pictures or videos showing the meaning of the terms or parts of the term and uploaded online for the student’s future reference.

2.3.4 Oral Anatomy and Function

*Course description*

The course will cover the nomenclature and function of the mouth, teeth and surrounding structures. Having this course in Module 2 will, in parallel, augment the student’s knowledge in oral anatomy and function since they will be learning this in the Japanese context as well. Thus, it will be easier for them to inter-relate terminology and function. Since oral anatomy is the basis of topics and situations in the dental profession, it is important for students to have a good foundation and understanding of it upon completion of this course. Topics should therefore include:

Types and structures of teeth (incisors, molars, enamel, dentin, pulp…)
Periodontium (periodontal ligament, alveolar bone, periodontitis)
Jaws (maxilla and mandible)
Oral Tissues (mucosa, gingiva, tongue)
Eruption Patterns (temporary and permanent teeth)
Tooth-Numbering Systems (ADA, ITDS, and Palmer’s Notation)
* ADA (American Dental Association); ITDS (International Two-Digit System)
Oral health and disease (oral hygiene, dental caries, caries assessment)
Dental Materials (types of materials/instruments, prosthetics)
Function (oral tissues, saliva, chewing, occlusion, speech)

*Delivery*

Teaching methods will include blended learning, etymology, slideshow presentations, and the use of model casts. Other teaching methods may be also adapted to meet the goals of the course. Information is basically presented through lecture since the course is a basic dental science. It is possible for non-dentist teachers to invite guest lecturers who are dentists in case the need arises. Practical activities may also be included especially in topics concerning types and structures of the teeth. Activities may include naming of teeth, identifying parts, or role playing of tooth type and function. Audiovisual aids that include clinical slides, videos, online sources and teaching model casts should be used to support classroom and practical activities.

2.3.5 English Conversation in Dentistry (Academic/Clinical Application)

*Course description*

Applied conversations regarding dental situations will constitute a major part of this course. The academic segment of the course will focus on communication between and among professionals and patients. Future dentists in Japan should be able to prepare themselves to meet, interact or collaborate with colleagues from other countries. Additionally, they should continue to have an aspiration for learning and sharing by reading recent scientific publications or

by presenting results of their experiences in international conferences. The course will include mentoring on how to read and write scientific papers, improve presentation skills, and foster research collaborations. The clinical segment will focus on patient-dentist communication, which is the verbal and non-verbal process through which a dentist obtains and shares information with a patient; and chair-side conversation exercises on dental consultation and treatment. The knowledge gained from basic and advanced terminology courses should be applied in this course.

*Delivery*

Applied conversation may be performed by groups through role playing. Since the focus is on dental situations, teachers should prepare a large number of case scenarios beforehand. Participants may act within their own groups or with other groups. Case scenarios should include a wide variety of situations including self-introduction to previously known and unknown colleagues, presenting a case study, answering inquiries by telephone, interviewing first-visit patients, medical/dental history-taking, scheduling recall visits, chair-side conversations, difficult-to-manage patients and pediatric patients. Students may be tasked to write dialogues for a case scenario they decide on and they can practice it amongst themselves or with other groups. It may be possible for non-native English teachers who do not have human resources for blended learning to invite guest native English teachers during the conversation classes. It is also possible to invite foreign researchers within the campus to interact with your students.

### 2.3.6 Computer-based Learning (E-Learning)

*Course description*

Learning in an electronically-supported environment is known to help in the implementation of the learning process. E-learning is essentially the computer and network-enabled transfer of skills and knowledge. Content is delivered via smart phones, internet, audio or video files, or through online databases. It can

be self-paced or instructor-led and includes media in the form of text, image, video and audio. If resources permit, adequate volumes of multimedia files, questionnaires, multiple-choice quizzes or electronic patient charts should be available for students. Computer-based activities integrated with practical or classroom-based situations have become increasingly popular in higher education and language learning. Teachers may set timeframes for content access and response or make the content freely available throughout the semester. Although the system can be beneficial, the set-up and maintenance of computers and device compatibility may require specialized staff.

*Delivery*

Private e-Learning platforms in Japan have collaborated with a number of universities in response to their specific academic needs. For instance, a system would include customizable reading, listening and writing exercises on a conversation between a tourist and a local. If the school is subscribed to such services, teachers should familiarize themselves with their content and applicability. Otherwise, teachers can design their own online tools using platforms that are available for free or those maintained by the university network. Teachers may design online quizzes for students to answer outside class or during their free time and in the same way, return results online. It is also possible for teachers to allow students to access print-outs of upcoming lectures so they will have an idea of the topics before the class. Additionally, an online discussion forum on case reports or a specific topic may be developed for students to prepare them for actual face-to-face discussions during class.

It is also highly recommended that electronic dental chart recording be included because most medical/dental institutes nowadays use electronic charts. Chart information will help students review and apply their knowledge on terminology, medical/dental conditions, tooth nomenclature and numbering, spellings, and writing skills (in diagnosis, prognosis or note-taking).

### 2.3.7 Peer Interaction (Advanced)

*Course description*

Since learning is social, at its best, it develops from interaction with others, as perceptions are shared, information is exchanged, and problems are solved. This course focuses on pairing or grouping students to perform specific tasks or roles for inter/intra-professional communication. The course will facilitate the improvement of critical thinking and discourse concerning dental topics, including dentist-patient and inter/intra-professional interaction through social interaction between their peers. The course aims to improve student's social behavior outcomes and demonstrable evidence of self-confidence in social interaction. The type of interaction in a second-language class usually depends on the teachers and most teachers would use a mix of activities to develop fluency and accuracy. In fluency-oriented activities, students should be able to speak without much interruption. This will encourage students to use as much of their language knowledge as they. In accuracy-oriented activities, students should be able to focus on the correct usage of grammar or vocabulary. Therefore, teachers should prepare a syllabus that would allot more time on peer-interaction activities and special seminars on professional growth such as speaking and presenting skills. Additionally, the school should be able to create a culture of dual language usage within the university not just among students but also among teachers in other departments.

*Delivery*

Group-work activities continue to comprise the advanced course. Scenarios on developing optimism, getting into a group, giving and receiving compliments, eliciting opinions, respecting differences, disagreeing politely, and building a positive reputation, are suggested. In succeeding activities, the use of simulated patients as part of the course will prepare students in their future dealings with actual English-speaking patients and colleagues. If possible, teachers should enforce an all English policy in the classroom. Teachers can create motivation in the form of interactive activities where the students need to

communicate in order to complete a task. For example, Student 1 has the full information of a case study concerning tooth decay and Student 2 needs to fill in the blanks. During their peer-to-peer discussions, it should be a basic and unalterable tenet that discussions should be in English and teachers, teaching assistants, or the students should circulate facilitating or checking the conversations. The final activity of Module 2 should be training for dentist-patient and inter/intra-professional communication using simulated patients. If possible, this should conform with the Japanese OSCE (Objective Structured Clinical Examination) standards with English as a means of communication and also universally accepted standards of inter/intra-professional communications and presentations in scientific meetings.

### 2.4 Learning Strategies

GIO and SBOs in syllabus design are complemented by Learning Strategies (LS) and Evaluation (EV). In simple terms, LS refer to methods that students use to learn. Weinstein and Mayer defined learning strategies broadly as "behaviors and thoughts that a learner engages in during learning" which are "intended to influence the learner's encoding process".[14] Later, Mayer specifically defined LS as "behaviors of a learner that are intended to influence how the learner processes information".[15]

### 2.5 Evaluation

Evaluation is the process through which teachers judge the quality of their own work or those of their students. The EVs of students' progress and acquisition of skills may include quizzes, classroom activities, assignments or any achievement towards mastering the objectives of the course. Since this core curriculum is intended for use in schools around Japan with different needs, LSs and EVs will be provided in the sample syllabus just for reference purposes. It will be the responsibility of the teachers to formulate their own LSs and EVs according to the priorities, preferences and situation of their respective schools and that of their students.

## UNIT 3: SAMPLE SYLLABUS

### 3.1 Module 1

3.1.1 One-Semester course

| Session | Topics | The student will be able to: |
|---|---|---|
| 1 | Introduction to Dental English | Understand why studying English is important to future dentists |
| 2 | Etymology | Understand the principles of the etymology of medical and dental terms |
| 3 | Introduction to Dentistry 1 | Have a knowledge on the history, scope and limitations of dentistry |
| 4 | Introduction to Dentistry 2 | Have a knowledge on the branches of dentistry and its nomenclature |
| 5 | Introduction to Dentistry 3 | Have a knowledge about the oral cavity, teeth, supporting structures and its nomenclature |
| 6 | Introduction to Dentistry 4 | Have a knowledge about the oral cavity, teeth, supporting structures and its nomenclature |
| 7 | Tooth-numbering systems | Classify all of the deciduous and permanent teeth according to the universal, Palmer & ITDS systems |
| 8 | Oral health and disease | Relate the role of the teeth to health and disease and vice versa, diagnosis and treatment |
| 9 | Oral health and disease | Relate the role of the teeth to health and disease and vice versa, diagnosis and treatment |
| 10 | Peer Interaction 1 (Beginners) | Tackle basic conversational tasks |
| 11 | Peer Interaction 2 (Beginners) | Tackle basic dentist-patient conversational tasks |
| 12 | Peer Interaction 3 (Beginners) | Tackle basic dentist-patient conversational tasks |
| 13 | Peer Interaction 4 (Beginners) | Tackle basic dentist-patient conversational tasks |
| 14 | Peer Interaction 5 (Beginners) | Tackle basic dentist-patient conversational tasks |
| 15 | Review | Review/master all lessons and terminology |
| 16 | Final Exams | Pass the course |

### 3.1.2 Two-Semester course

First Semester:

| Session | Topics | The student will be able to: |
|---|---|---|
| 1 | Introduction to Dental English | Understand why studying English is important to future dentists |
| 2 | Etymology (Lecture) | Understand the principles of the etymology of medical and dental terms |
| 3 | Etymology (Exercise) | Apply the principles of the etymology of medical and dental terms through word exercises |
| 4 | Introduction to Dentistry 1 | Have a knowledge on the history, scope and limitations of dentistry |
| 5 | Introduction to Dentistry 2 | Have a knowledge on the branches of dentistry and its nomenclature |
| 6 | “ | Have a knowledge on the branches of dentistry and its nomenclature + Review |
| 7 | Introduction to Dentistry 3 | Have a knowledge about the oral cavity, supporting structures and its nomenclature |
| 8 | “ | Have a knowledge about the oral cavity, supporting structures and its nomenclature + Review |
| 9 | Tooth-numbering systems | Classify all of the deciduous and permanent teeth according to the universal, Palmer, and ITDS systems |
| 10 | “ | Universal, Palmer, and ITDS systems |
| 11 | Oral Health and Hygiene | Gain knowledge on oral health and good oral hygiene maintenance |
| 12 | Oral Diseases (Tooth Decay) | Gain knowledge about tooth decay, its cause, process and treatment |
| 13 | “ | “ |
| 14 | “ | “ |
| 15 | Review | Review/master all lessons and terminology |
| 16 | Final Examination | Pass the course |

3.1.2 Two-Semester course

Second Semester:

| Session | Topics | The student will be able to: |
|---|---|---|
| 17 | Review | Recall topics and terminology from the previous semester (Semester One) |
| 18 | Dental Materials | Be familiar with common equipment and materials used in dentistry |
| 19 | Oral Diseases (Soft Tissues) | Gain knowledge on common oral diseases affecting soft tissues (gingiva, periodontium, mucosa) |
| 20 | Diagnosis and Treatment | Know the basis for diagnosis and treatment of oral diseases |
| 21 | E-Learning | Experience computer-based learning (Vocabulary) |
| 22 | E-Learning | Experience computer-based learning (Electronic Dental Charting) |
| 23 | Medical/Dental Conversation | Understand the basic concepts of conversation in a medical or dental setting |
| 24 | Peer Interaction 1 (Beginners) | Tackle basic conversational tasks |
| 25 | “ | “ |
| 26 | “ | “ |
| 27 | Peer Interaction 2 (Beginners) | Tackle basic dentist-patient conversational tasks |
| 28 | “ | “ |
| 29 | “ | “ |
| 30 | “ | “ |
| 31 | Review | Review/master all lessons and terminology |
| 32 | Final Examination | Pass the course |

### 3.2 Module 2

3.2.1 One-Semester course

| Session | Topics | The student will be able to: |
|---|---|---|
| 1 | Introduction | Have an idea of the topics, schedules and requirements of the course |
| 2 | Principles of Etymology | Understand the principles of the etymology of medical and dental terms |
| 3 | Terminology | Recall, decipher and construct medical/dental terms |
| 4 | Oral Anatomy | Identify tooth types and, morphology |
| 5 | " | Master tooth-numbering systems and eruption patterns |
| 6 | Oral Physiology | Understand the physiology of the mouth, teeth, saliva |
| 7 | " | " |
| 8 | English in Dentistry (Academic) | Know the implications of reading, listening, writing, and speaking English for academic purposes |
| 9 | English in Dentistry (Clinical) | Know the implications of reading, listening, writing and speaking English for professional purposes |
| 10 | E-Learning | Experience computer-based learning (Vocabulary) |
| 11 | E-Learning | Experience computer-based learning (Electronic Dental Charting) |
| 12 | Peer Interaction 1 (Advanced) | Tackle basic and complex conversational tasks |
| 13 | Peer Interaction 2 (Advanced) | Tackle basic and complex dentist-patient conversational tasks |
| 14 | Peer Interaction 3 (Advanced) | Tackle basic and complex inter/intra-profession conversational tasks |
| 15 | Peer Interaction 4 (Advanced) | Tackle basic and complex inter/intra-profession conversational tasks |
| 16 | Final Examination | Pass the course |

3.2.2 Two-Semester course

First Semester:

| Session | Topics | The student will be able to: |
|---|---|---|
| 1 | Introduction | Have an idea of the topics, schedules and requirements of the course |
| 2 | Principles of Etymology | Understand the principles of the etymology of medical and dental terms |
| 3 | Review of Terminology | Recall, decipher and construct medical/dental terms |
| 4 | Oral Anatomy | Identify tooth types, morphology and function |
| 5 | “ | “ |
| 6 | Tooth-numbering systems | Classify all of the deciduous and permanent teeth according to the universal, Palmer & ITDS systems |
| 7 | “ | Universal, Palmer, and ITDS systems + Review |
| 8 | Dental Materials | Be familiar with common equipment and materials used in dentistry |
| 9 | Oral Health and Hygiene | Gain knowledge on oral health and good oral hygiene maintenance |
| 10 | Oral Diseases (Tooth Decay) | Gain knowledge about tooth decay, its cause, process and treatment |
| 11 | “ | “ |
| 12 | Oral Diseases (Soft Tissues) | Gain knowledge on common oral diseases affecting soft tissues (gingiva, periodontium, mucosa) |
| 13 | Diagnosis and Treatment | Know the basis for diagnosis and treatment of oral diseases |
| 14 | Diagnosis and Treatment | Know the basis for diagnosis and treatment of oral diseases |
| 15 | Review | Review/master past lessons |
| 16 | Final Examination | Pass the course |

3.2.2 Two-Semester course

Second Semester:

| Session | Topics | The student will be able to: |
|---|---|---|
| 17 | Review | Recall topics and terminology from the previous semester (Semester One) |
| 18 | English in Dentistry (Academic) | Know the implications of reading, listening, writing and speaking English for academic purposes |
| 19 | English in Dentistry (Clinical) | Know the implications of reading, listening, writing and speaking English for professional purposes |
| 20 | E-Learning | Experience computer-based learning (Vocabulary) |
| 21 | “ | “ |
| 22 | E-Learning | Experience computer-based learning (Electronic Dental Charting) |
| 23 | “ | “ |
| 24 | Peer Interaction 1 (Advanced) | Tackle basic and complex conversational tasks |
| 25 | “ | “ |
| 26 | Peer Interaction 2 (Advanced) | Tackle basic and complex dentist-patient conversational tasks |
| 27 | “ | “ |
| 28 | Peer Interaction 3 (Advanced) | Tackle basic and complex inter-profession conversational tasks |
| 29 | “ | “ |
| 30 | Peer Interaction 4 (Advanced) | Tackle a role playing scenario between an English-speaking simulated patients and students |
| 31 | “ | “ |
| 32 | Final Examination | Pass the course |

### 3.3 Sample Syllabus with LS and EV

**Module 1** (One-Semester course)

| Session | Topics | Learning Strategies<br>Students will… | Evaluation |
|---|---|---|---|
| 1 | Introduction to Dental English | understand why studying English is important to future dentists | 60% of Final Exams |
| 2 | Etymology | understand the principles of the etymology of medical/dental terms | |
| 3 | Introduction to Dentistry 1 | know history, scope and limitations of dentistry | |
| 4 | Introduction to Dentistry 2 | Know the branches and nomenclature of dentistry | |
| 5 | Introduction to Dentistry 3 | know the oral cavity and the teeth | |
| 6 | Introduction to Dentistry 4 | know the oral cavity and the teeth | |
| 7 | Tooth-numbering systems | classify correctly deciduous and permanent teeth according to the universal, Palmer & ITDS systems | |
| 8 | Oral health and disease | relate the role of the teeth to health and disease and vice versa, diagnosis and treatment | |
| 9 | Oral health and disease | relate the role of the teeth to health and disease and vice versa, diagnosis and treatment | |
| 10 | Medical Interview | know the basics of patient-doctor communication and medical/dental interviewing | 40% of Final Exams |
| 11 | Peer Interaction 1 (Beginners) | tackle basic conversational tasks through pair or group-work, blended learning | |
| 12 | Peer Interaction 2 (Beginners) | tackle basic conversational tasks through pair or group-work, blended learning | |
| 13 | Peer Interaction 3 (Beginners) | tackle basic conversational tasks through pair or group-work, blended learning | |
| 14 | Peer Interaction 4 (Beginners) | tackle basic conversational tasks through pair or group-work, blended learning | |
| 15 | Review | master all lessons and terminology through a comprehensive review with class participation | |
| 16 | Final Examination | have understood the importance of dental English by passing it | 60% or higher passing grade |

**Module 2** (One-Semester course)

| Session | Topics | Learning Strategies<br>Students will… | Evaluation |
|---|---|---|---|
| 1 | Introduction | have an idea of the topics and requirements of the course | 40% of Final exams |
| 2 | Principles of Etymology | understand the principles of the etymology of medical/dental terms | |
| 3 | Terminology | recall, decipher and construct medical/dental terms | |
| 4 | Oral Anatomy | identify tooth types and morphology | |
| 5 | “ | master tooth-numbering systems and eruption patterns | |
| 6 | Oral Physiology | understand the physiology of the mouth, teeth, and saliva | |
| 7 | “ | “ | |
| 8 | English in Dentistry (Academic) | improve reading, listening, writing, & speaking English for academic purposes | 60% of Final exams |
| 9 | English in Dentistry (Clinical) | improve reading, listening, writing & speaking English for clinical purposes, medical/dental interview | |
| 10 | E-Learning | Experience computer-based learning (Vocabulary) | |
| 11 | E-Learning | Experience computer-based learning (Electronic Charting) | |
| 12 | Peer Interaction 1 (Advanced) | tackle basic dentist-foreign dentist conversational tasks through pair or group work, simulation | |
| 13 | Peer Interaction 2 (Advanced) | tackle basic dentist-patient conversational tasks through pair or group work, simulation | |
| 14 | Peer Interaction 3 (Advanced) | tackle basic dentist-patient conversational tasks through pair or group work, simulation | |
| 15 | Peer Interaction 4 (Advanced) | tackle basic dentist-patient conversational tasks through pair or group work, simulation | |
| 16 | Final Examination | have understood the importance of dental English by passing it | 60% or higher passing grade |

## UNIT 4: IMPLEMENTATION

Curriculum implementation is the practical application of courses, subjects and activities prescribed to help students acquire knowledge and experience. However, it is not the endpoint and as such, should be evaluated regularly. Globalization and the ever-changing needs of students, dentists and patients require competent professionals with exceptional vision, knowledge, technical and communication skills. Future Japanese dentists must be able to work to readily and confidently provide optimal care and instruction to both Japanese and international patients. They should also be able to see themselves as partners in the international community of dental professionals as well.

The implementation of this core curriculum for dental English courses is only meant to guide curriculum developers as to the basic and current needs that have to be addressed as far as English education in dental schools are concerned. The core curriculum was developed based on a collection of teaching techniques and experiences of teachers of the course, the current needs of dental students, and a positive outlook for a future Japanese dentist who is globally-competitive. With the approval of the Ministry of Education, Culture, Sports, Science and Technology of Japan, the core may be revised and improved freely as long as the basic principles stated in this report are adhered to. Teachers of the course/s become the ultimate implementers and are therefore very important in the implementation process. They are free to choose or mix various aspects of recommended courses, topics or exercises as appropriately as possible. Core curriculum implementation therefore refers to how the officially designed course of study is translated by the teacher into their own syllabi and delivered to students. This translation process is then evaluated regularly by the teachers or teachers and students for further improving the course.

As a personal note, I propose that the core curriculum that took 3 years to develop be implemented for at least two semesters. As with OSCE, students who were able to experience medical interview during their 3$^{rd}$ year are more likely to be confident later.

## UNIT 5: EVALUATION

Evaluation of the curriculum is usually based on the students' and teachers' assessment of the course, teaching or learning resources and the overall learning environment (Example: creating a dual-language culture within the dental school). This is because of the constant changes in academic, research, social, economic and technological aspects of society. The Commonwealth of Learning (October 2000) suggested ways for teachers to evaluate a course curriculum and one way involves the collection of descriptive and judgmental information for the purpose of establishing whether courses, topics or exercises are doing what they are expected to do. Another way involves comparing the performance of one or more students with set standards. Such an evaluation determines the extent to which the objectives of a learning activity are being realized. Another way is concerned with the identification of deficiencies in an educational program or syllabus for the purpose of effecting revision and improvement. It is advised to note that curriculum evaluation exercises usually combine these three activities. Data is collected for passing judgment, to identify deficiencies in programs and to analyze programs in order to determine alternatives or find appropriate interventions.

Evaluation and re-evaluation is said to occur in levels starting with the school level, cluster level, regional level and national level. At the school level, teachers take note about concerns, materials and problems they and the students face. If there are two or more teachers of the course (including teaching assistants), they should meet and discuss these concerns. Evaluation in the regional level is done through discussions by schools that are geographically close to each other in terms of prefectural limits while evaluation done in the national level is done through discussions by teachers of the course and education officials. Teachers of dental English courses should therefore take the responsibility of constantly assessing the course and reporting their findings to the succeeding levels. The cycle of discussing, addressing, evaluating and implementing the curriculum is a continuous process and all sectors should be represented well during each cycle.

## UNIT 6: REFERENCES


1. Rodis O, Matsumura S, Kariya N, et al. Undergraduate dental English education in Japanese dental schools. J Dent Educ 2013;77(5):656-663.

2. Ministry of Education, Culture, Sports, Science, and Technology. The course of study for secondary school. 1989; Tokyo.

3. Ministry of Education, Culture, Sports, Science, and Technology. Regarding the establishment of an action plan to cultivate Japanese with English abilities. 2003.

4. Takazoe I. Recent changes in the dental curriculum in Japan. Int Dent J 1998;38(4):252-4

5. Komabayashi T, Raghuraman K, Raghuraman R, Toda S, Kawamura M, Levine S, et al. Dental Education in India and Japan: Implications for US Dental Programs for Foreign-Trained Dentists. J Dent Educ 2005;69(4):461-9.

6. Morse Z, & Nakahara S. English language education in Japanese dental schools. Eur J Dent Educ 2001;5:168–172.

7. McVeigh BJ. Japanese higher education as myth 2002; London: ME Sharpe.

8. Stapleton P. Japanese Universities: Change or risk marginalization. The Language Teacher 2011;35(5):37-41.

9. Daily Yomiuri. English-language University breaks mold. The Daily Yomiuri, December 14, 2004:16. Japan.

10. The Science Council of Japan. The prospects of dentistry in Japan. Retrieved: May 2011; http://www.scj.go.jp/ja/info/kohyo/pdf/kohyo-21-h-2-8.pdf.

11. Telloyan J, Iwata J, Iga M. English Education as seen by Japanese Doctors, Researchers and Students. Bull Shimane Univ Fac Med 2009;32:7-12.

12. Rodis O, Kariya N, Nishimura M, Matsumura S, Tamamura R. Needs Analysis: Dental English for Japanese Dental Students. Asian EFL Journal October 2011;55:1-20.

13. Rodis O, Kariya N, Nishimura M, Matsumura S. The student-teacher shuttle card for Japanese dental students taking a dental English course. TESOL Journal March 2010; 2(1):73-90.

14. Weinstein CE, and Mayer RE. (1986; p. 315). The teaching of learning strategies. In M. Wittrock (Ed.), Handbook of research on teaching (pp. 315-327). New York, NY: Macmillan.

15. Mayer R. (1988; p. 11). Learning strategies: An overview. In C Weinstein, E Goetz and P Alexander (Eds.), Learning and Study Strategies: Issues in Assessment, Instruction, and Evaluation (pp. 11-22). New York: Academic Press.

## UNIT 7: APPENDICES

- Survey for Dental Schools
- Current Status of Dental English Education
- Current Distribution of Dental English Courses
- The 1st Meeting Brochure and Program
- The 2nd Meeting Brochure and Program
- The 1st Meeting Interim Report
- The 2nd Meeting Interim Report

平成 23 年 2 月 23 日

歯学部教務担当係長殿

岡山大学大学院医歯薬学総合研究科
歯学部学部長
松尾龍二

歯学部臨床歯科英語教育に関するアンケートへの協力について（依頼）

拝啓　時下清祥のこととお慶び申し上げます。

近年，歯学部学生英語能力の向上が必要とされていることについては論を俟たないところでありますが，本学では効果的な歯学部英語教育のため新たな歯科英語のカリキュラム開発を計画いたしております。

本学ではこれまで，歯学部学生歯科英語教育については，英語を主とする外国人の文部教官が英語を用いて口腔内各部の名称，歯科材料や診療室での英会話の講義を行ってきました。

しかし，昨今の社会状況や国際化，学生の学習意欲などを鑑みるに「もっと使える英語を学生にもっと楽しく」と歯科英語教育に携わる現場教員からの提案があり，この歯科英語のカリキュラム開発が計画されました。

この度，他大学での歯科英語教育の現状を知りたく，全国２９大学の現状を調査することになりました。

つきましては，貴大学での歯科英語教育の現状を教えていただきたいと存じます。

大変お忙しい中，誠に恐縮ですが標記アンケートへの御回答いただきたく御協力の程，よろしくお願い申し上げます。

なお，御不明な点がございましたら，下記までお問い合わせください。

敬具

記

１．御回答方法について

御回答方法につきましては，今回，ＦＡＸにて御送付お願い致します。

(FAX番号:086-235-7564)岡山大学医歯薬総合研究科等学務課教務グループ歯学部担当

２．御回答期限

大変恐縮に存じますが，平成２３年３月２０日までに御回答いただけますようお願いたします。

３．お問い合わせ先

お問い合わせは，お手数ですが電子メールにてお願いいたします。早急に回答いたします。

国立大学法人　岡山大学大学院医歯薬学総合研究科歯学英語担当
ロディス・オマー　omarodis@md.okayama-u.ac.jp

以上

February 23, 2010

The Student Affairs Office
Okayama University Dental School

Dear Sir/Madam,

In 1999, a survey was conducted by Zac Morse and Sen Nakahara of the Nippon Dental University, Niigata, Japan regarding English language education in Japanese Dental Schools. Shigaku Eigo, or Dental English was introduced into the dental curriculum to address English learning and communication needs of dental students. More than 10 years have passed and there is still no consensus concerning what the course comprises. For the past 3 years, I have taught the course to first year students of Okayama University and I have always been concerned of the fact that it is only offered for two quarters during the 1st of 6 years of dental school. This is not enough! For future dentists of Japan, there is a need to develop a core curriculum for the Dental English course to enable future dentists to remain globally competitive and well-informed.

In this regard, I am in the process of developing a core curriculum based on academically-approved course context and a needs-analysis of dental students. With this, I have included some pertinent questions from the Morse/Nakahara survey for updated information. I hope that the information gathered will be instrumental in improving the course and dental needs of students.

Thank you for your cooperation.

Respectfully yours,

Omar M.M. Rodis, DDM, MaHSM, PhD
Assistant Professor
Department of Behavioral Pediatric Dentistry
Okayama University Graduate School of Medicine, Dentistry & Pharmaceutical Sciences
2-5-1 Shikata-cho, Okayama City, Kita-ku, 700-8525

（和訳）1999 年に日本歯科大学新潟校で歯科大学（歯学部）の英語教育に関する調査がザック モースと中原　泉先生によって行なわれました。歯学英語もしくは Dental English は，歯科学生の英語学習とそのコミュニケーション能力の必要性に鑑みて，歯学教育のカリキュラムに組み入れられました。そして 10 年以上が経過しました。しかし，歯科英語教育に関するコンセンサスはまだ得られておりません。過去 3 年間，私は岡山大学歯学部の一年生に歯学英語を教えました。それは６年間の歯学部教育の最初の１年間の 4 分の 2（クオーター制なので）の期間だけで，私はその期間がいつも短く感じています。これは十分ではありません。今こそ日本の将来の歯科医師のために，日本の歯科医師が国際的に競争でき知性的職業であり続けるために歯科英語の授業のためのコアカリキュラムを開発する必要があります。この件については，私はこれまでに学問的に認められた授業の状況と歯科医学生のニーズに応じた分析に基づくコアカリキュラムを開発しているところです。

今回のアンケートでは，私はあたらしく情報を収集するためモース/中原調査の中から，いくらかのそれらに関連する質問を含めました。私は，もちろん今回集められた情報が歯科英語の授業と学生の歯科分野におけるニーズの向上に役立つことを望みます。

ご協力よろしくお願いいたします。

敬具

ロディス・オマー
岡山大学大学院医歯薬学総合研究科
行動小児歯科学分野　助教

# Please fax completed form to: **(086) 235-7564**

岡山大学医歯薬総合研究科等学務課教務グループ歯学部担当

**Notice** This survey will be used to assess the current situation of teaching a dentistry-related English course among the 29 dental schools in Japan. The subject of assessment is the Dental English course, also known as Shigaku Eigo. The course should be differentiated from general English courses also offered in the dental curriculum.

**御注意** この調査は，日本全国２９校の歯科大学（歯学部）の中で歯科英語の授業の現状を調べるために行っています。調査の対象は，一般的に「歯学英語」といわれる授業です。大学での一般教養としての「英語」の授業ではありません。

大学の名前 国立 私立

**Name of School**: ________________________________________________ [Public・Private]

**1. Do you offer the Dental English course?** Yes ・ No

歯学英語の授業を行っていますか

**2. When is it offered (Year level and semester)?**

1st yr ・ 2nd yr ・ 3rd yr ・ 4th yr ・ 5th yr ・ 6th yr

いつですか 「何年生・前期後期」 前 後 ・ 前 後 ・ 前 後 ・ 前 後 ・ 前 後 ・ 前 後

**3. Number of minutes per class?** __________

授業は何分間ですか

**4. Average number of students per class?** __________

授業の受講学生の人数は何人ですか

**5. Number of Dental English Educator/s?** __________

歯学英語を担当している先生の人数は何人ですか

**6. Background of Educator/s?**

歯学英語を担当している先生の教育者としての背景はどのような先生方でしょうか

Linguist ・ Health-related ・ Dentist ・ Others

言語学者 ・ 健康関連（医師・看護師）・ 歯科医師等 ・ その他

**7. Use of official textbook/s?** Yes: (教科書名)___________________________________ ・ No

決められた教科書を使用しますか

**8. Offer elective Dental English education to postgraduate students?** Yes ・ No

**大学院生に（例えば選択科目のような形ででも）歯科英語の教育を行っていますか**

for dental schools in Japan/日本の歯科大学（大学歯学部）用

## The current status of dental English courses taught in Japan's 29 dental schools (as of March 2011: updated)

| | School | Offer Course | When Offered ($\text{Year}^{\text{Semester}}$) | Class Hours (minutes) | Ave. # of Students | # of Teachers | Background of Teacher | Offer to Postgrads | Textbook |
|---|---|---|---|---|---|---|---|---|---|
| 1 | Hokkaido University | no | x | x | x | x | x | yes | x |
| 2 | Tohoku University | yes | $5^{1}$ | 50 | 55 | 11 | others | yes | x |
| 3 | Tokyo Medical and Dental University | yes | $1^{2}$ $2^{1\ 2}$ $3^{1}$ $4^{1}$ $5^{1}$ | 90/50 | 55 | 4 | linguist/dentist | no | None |
| 4 | Niigata University | yes | $3^{1\ 2}$ $4^{2}$ | 90 | 40 | 1 | dentist | yes | Dental Chair-side Communication, etc. |
| 5 | Osaka University | no | x | x | x | x | x | yes | x |
| 6 | Okayama University | yes | $1^{2}$ | 90 | 52 | 2 | dentists | no | None |
| 7 | Hiroshima University | yes | $3^{1\ 2}$ $4^{2}$ | 90 | 55 | 16 | dentists/others | yes | None |
| 8 | Tokushima University | yes | $3^{2}$ | 60 | 45 | 20 | dentist | no | Language of Medicine |
| 9 | Kyushu Dental College | yes | $3^{2}$ $5^{1}$ | 90 | 87 | 2 | others | no | Effective Academic Writing |
| 10 | Kyushu University | yes | $1^{1\ 2}$ $2^{2}$ $3^{1\ 2}$ $4^{1\ 2}$ | 90 | 60 | 2 | linguist/dentist | yes | Kyushu University Dental English Series |
| 11 | Nagasaki University | yes | $1^{2}$ | 180 | 50 | 2 | dentists | no | None |
| 12 | Kagoshima University | no | x | x | x | x | x | no | x |
| 13 | Health Sciences University of Hokkaido | yes | $2^{2}$ $3^{1}$ $5^{1}$ | 80 | 60 | 1 | health | no | Concise Human Body |
| 14 | Iwate Medical University | yes | $2^{1\ 2}$ $3^{1}$ $4^{1\ 2}$ | 90 | 80 | 15 | ling/dent/others | yes | A Way to Good Health |
| 15 | Ohu University | yes | $1^{1\ 2}$ $2^{1\ 2}$ | 60 | 100 | 2 | linguist/others | yes | None |
| 16 | Meikai University | yes | $1^{1\ 2}$ $2^{1\ 2}$ $3^{1}$ $4^{1}$ $6^{1\ 2}$ | 90 | 120 | not stated | dentist/others | no | Yes (not stated) |
| 17 | Nihon University Matsudo | no | x | x | x | x | x | yes | x |
| 18 | Tokyo Dental College | yes | $2^{1}$ $3^{1}$ | 90 | 98 | 1 / 14 | linguist/dentist | no | Yes (not stated) |
| 19 | Nippon Dental University Tokyo | yes | $2^{1\ 2}$ | 80 | 147 | 2 | others | no | Yes (not stated) |
| 20 | Nihon University School of Dentistry | yes | $2^{1\ 2}$ $3^{1}$ | 50 | 80 | 2 / 6 | linguist/dentist | no | None |
| 21 | Showa University | yes | $2^{1\ 2}$ $3^{1}$ | 90 | 30 | 7 | others | yes | Speaking of Speech |
| 22 | Tsurumi University | yes | $2^{1\ 2}$ | 85 | 38 | 4 | dentist/others | yes | Oxford English for Career Med |
| 23 | Kanagawa Dental College | yes | $2^{2}$ $3^{1}$ | 75 | 95 | 1 | others | yes | None |
| 24 | Nippon Dental University - Niigata | yes | $1^{1\ 2}$ $2^{1\ 2}$ | 80 | 70 | 2 | linguist | no | Understanding Dentistry |
| 25 | Matsumoto Dental University | yes | $1^{1\ 2}$ $2^{1\ 2}$ | 90 | 50 | 1 | others | yes | Access to Simple English |
| 26 | Asahi University | yes | $1^{1\ 2}$ | 90 | 75 | 1 | others | no | Yes (not stated) |
| 27 | Aichi Gakuin University | no | x | x | x | x | x | no | x |
| 28 | Osaka Dental University | no | x | x | x | x | x | no | x |
| 29 | Fukuoka Dental College | no | x | x | x | x | x | no | x |

**Current distribution of dental English education per year level** (For the 22 schools offering the course: updated)

| | 1st year | | 2nd year | | 3rd year | | 4th year | | 5th year | | 6th year | |
|---|---|---|---|---|---|---|---|---|---|---|---|---|
| | 1st sem | 2nd sem | 1st sem | 2nd sem | 1st sem | 2nd sem | 1st sem | 2nd sem | 1st sem | 2nd sem | 1st sem | 2nd sem |
| **Public**<br>(25 course slots) | ● | ●●●● | ● | ●● | ●●●● | ●●●●● | ●● | ●●● | ●●● | | | |
| **Private**<br>(44 course slots) | ○○○○○ | ○○○○○ | ○○○○○<br>○○○○○ | ○○○○○<br>○○○○○<br>○ | ○○○○○<br>○○ | | ○○ | ○ | ○ | | ○ | ○ |
| **All**<br>(69 course slots) | ●○○○○<br>○ | ●●●●○<br>○○○○ | ●○○○○<br>○○○○○<br>○ | ●●○○○<br>○○○○○<br>○○○ | ●●●●○<br>○○○○○<br>○ | ●●●●● | ●●○○ | ●●●○ | ●●●○ | | ○ | ○ |

● Public schools
○ Private schools

# The 1st Discussion Forum to Develop a Core Curriculum for Dental English Education

## 日本の歯科大学における歯科英語教育コアカリキュラム開発に関してのディスカッションフォーラム

August 28, 2011 9:00 AM – 5:00 PM

Okayama International Center, Okayama City, Japan

| Participating Schools | Name of Representative/s |
|---|---|
| | |
| Hokkaido University 北海道大学 | Taro Arima |
| Health Sciences University of Hokkaido 北海道医療大学 | Yujiro Handa |
| Iwate Medical University 岩手医科大学 | Yoshinori Sahara / James Hobbs |
| Tokyo Medical & Dental University 東京医科歯科大学 | Naoko Seki |
| Tokyo Dental College 東京歯科大学 | Yoshiaki Shibaie |
| Nippon Dental University Tokyo 日本歯科大学生命 | Chie Yanai |
| Showa University 昭和大学 | Masaki Ohno |
| Kanagawa Dental College 神奈川歯科大学 | Martin Peters |
| Niigata University 新潟大学 | Roxana Stegaroiu |
| Nippon Dental University Niigata 日本歯科大学新潟生命 | Ikuo Kageyama |
| Asahi University 朝日大学 | Hironori Tsuchiya |
| Aichi Gakuin University 愛知学院大学 | Kazuyoshi Suzuki |
| Osaka Dental University 大阪歯科大学 | Junichi Fujita |
| Okayama University 岡山大学 | Omar Rodis / Seishi Matsumura / Naoyuki Kariya / Toshiko Yoshida |
| Hiroshima University 広島大学 | Hiroko Oka |
| Kyushu University 九州大学 | Jane Harland |
| Nagasaki University 長崎大学 | Etsuko Watanabe |
| | |

## Program:

| | |
|---|---|
| 09:00-09:10 | Welcome Remarks |
| 09:10-09:40 | Background & Objectives |
| 09:40-12:00 | Discussion Forum 1 (Current Status of Dental English) |
| 12:00-13:00 | Lunch Break |
| 13:00-16:00 | Discussion Forum 2 (Proposed Dental English Curriculum) |
| 16:00-16:50 | Recap & Other Matters |
| 16:50-17:00 | Closing Remarks |

**Host**: Okayama University
**Contact Person**: Omar Marianito Maningo Rodis
Tel: 086 235-6716 / 235-6717
Fax: 086 235-6719
Cell: 080 4260-8248
email: omarodis@md.okayama-u.ac.jp

Supported by a grant from the Ministry of Education, Culture, Sports, Science and Technology, Japan [23531201]

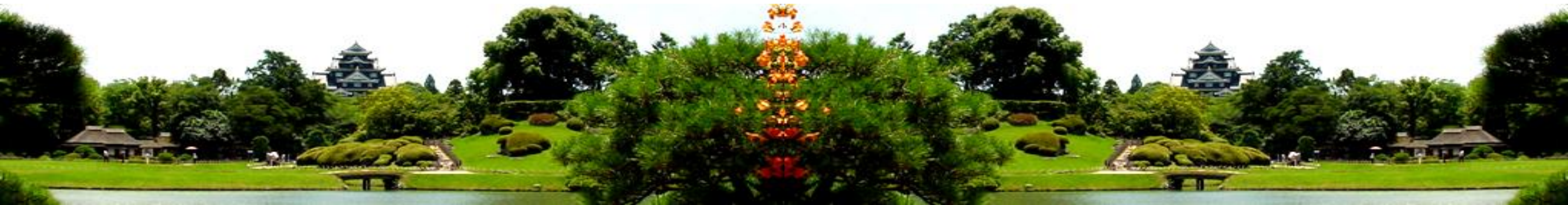

# Agenda

**Morning Session:**

1. Summary of current status of Dental English among Japan's dental schools and student's competency. (Rodis)
2. Short comments or discussion on these syllabi implementation, teaching/learning methods. (Participants)

**Afternoon Session** (Group work)**:**

1. Strategies of respective teachers on applying English medico-dental terminologies to the undergraduate dental course.
2. Agreement on "GIO (General Instructional Objectives) and SBOs (Specific Behavioral Objectives)" of at least 2 or 3 years' courses.
3. Feasibility of offering the course as a compulsory dental subject.
4. Possibility of offering the course for 6 years, or if not, deciding the most feasible.
5. The feasibility of including Dental English in the National Dental Board Exams.
6. What is the specific objective in teaching English including medico-dental terms to undergraduate dental students?
7. Whether proficiency of English among undergraduate students is an important topic for dental faculty?
8. Do students have sufficient exposure to English media, textbook and references in their daily activities?
9. Do students have good exposure to oral communication in English in their study and training processes?
10. How basic medical/dental subjects utilize English together with Japanese terms in the classrooms and laboratory.

**Other Matters**:

1. Discuss plans for the 2nd meeting.
2. MEXT representation
3. Discuss the possibility of organizing a support group or a society for dental English education.

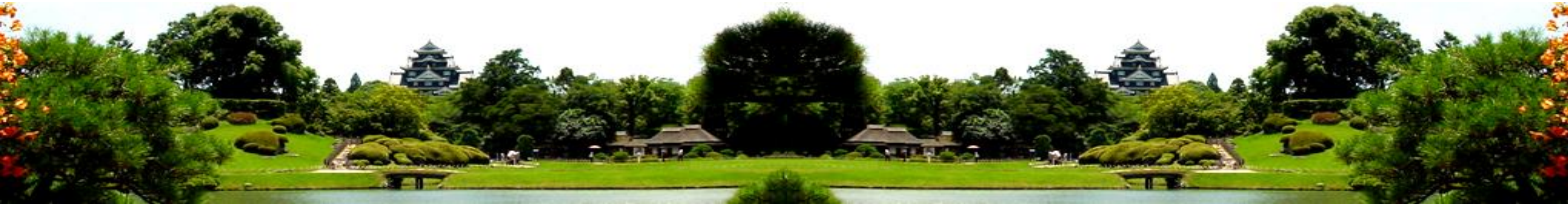

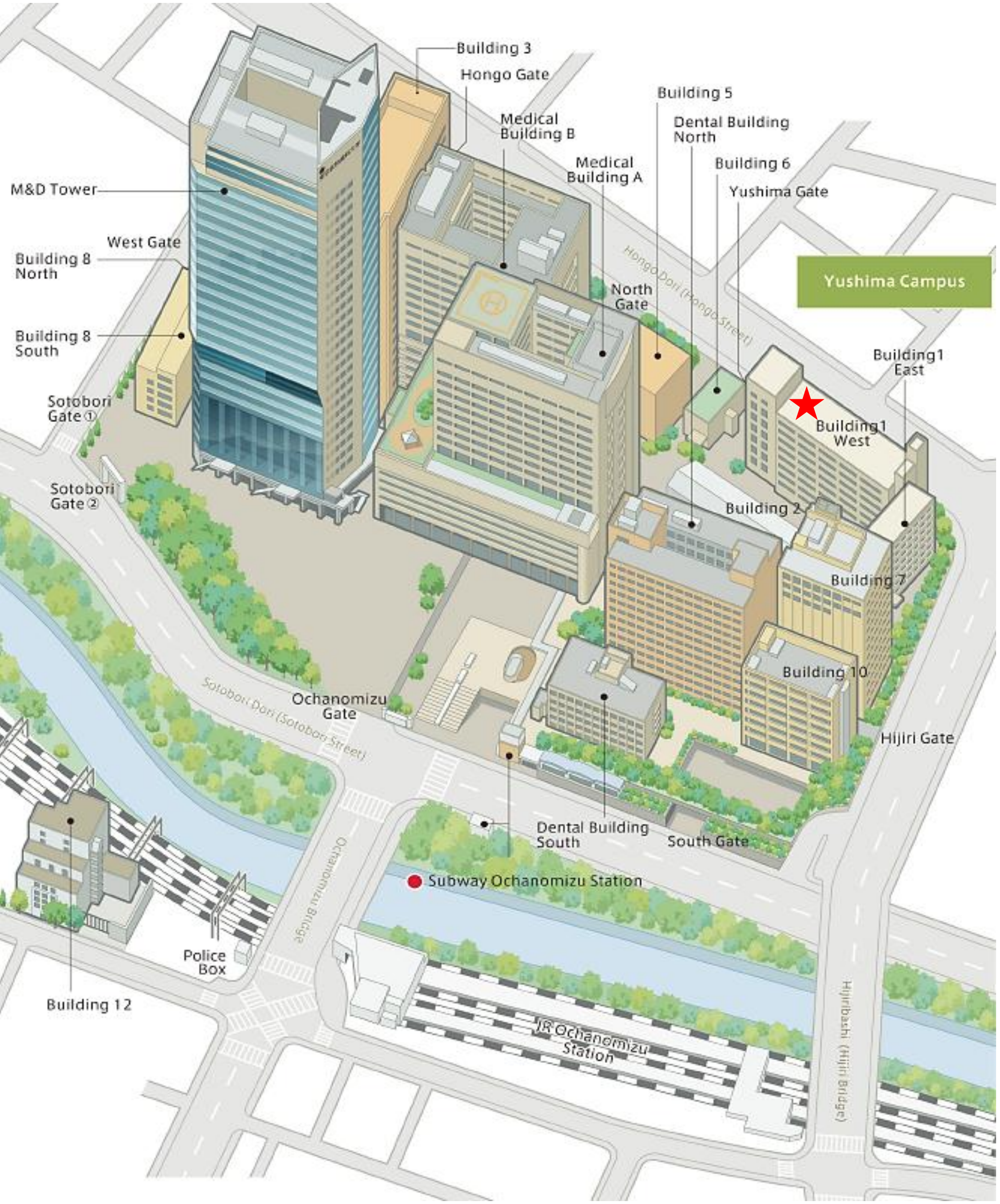


★ 6F Building 1 West, Yushima Campus「東京医科歯科大学湯島地区1号館西6F」

# The 2nd Discussion Forum to Develop a Core Curriculum for Dental English Education

# 日本の歯科大学における歯科英語教育コアカリキュラム開発に関するディスカッションフォーラム（第２回）

6F Building 1 West, Yushima Campus「東京医科歯科大学湯島地区1号館西6F」

Tokyo Medical and Dental University, Tokyo, Japan

June 9, 2012　10:00 AM – 6:00 PM

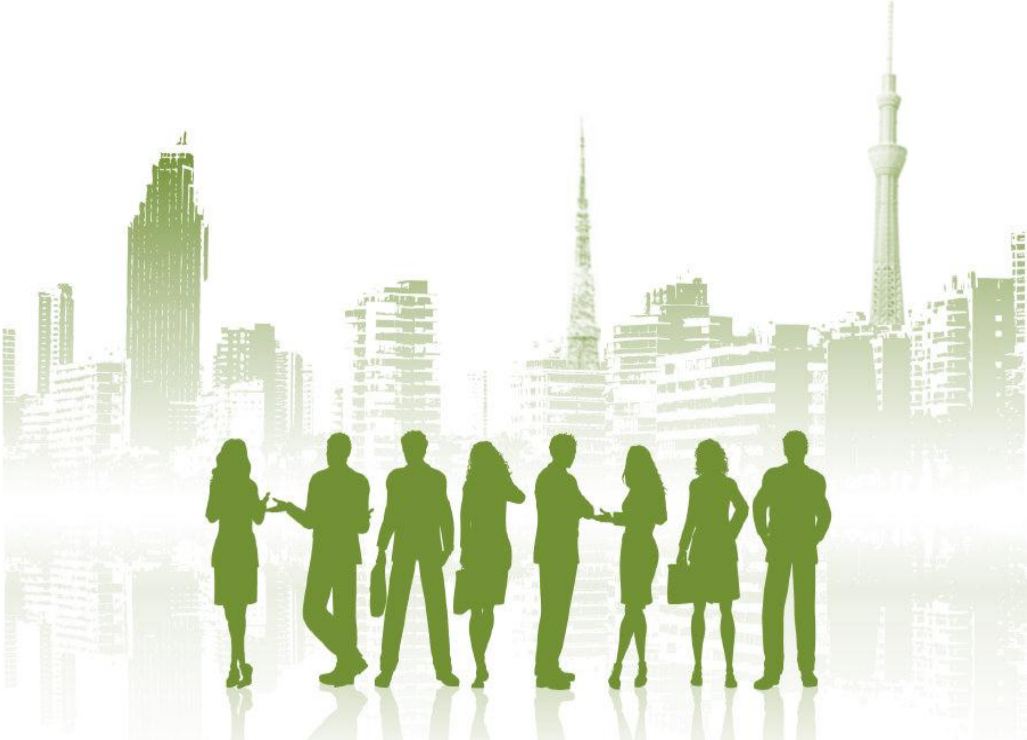

Supported by a grant from the Ministry of Education, Culture, Sports, Science and Technology, Japan [23531201]

# Message

The 1st meeting, a discussion forum, proved to be a successful gathering of educators responsible for dental English education. Their presentations on the current status of English courses in dentistry and the fact-finding discussions proved to be a good source of base knowledge for the development of a core curriculum. Although the discussions covered a variety of issues, most of it focused on the importance of motivation and dental English terminology as basic requirements in preparing students to communicate confidently in English with international patients and colleagues. The presentations of participants on how this was implemented in their respective schools provided insights to other participants as well.

The 2nd meeting will facilitate a seminar and deliberation to draft a core curriculum that focuses on patient-dentist communication and inter-profession communication. There will be guest speakers from the Tokyo Medical University's Department of International Medical Communications (DIMC) talking about topics concerning the development and implementation of medical English courses.

On behalf of my co-investigators, I sincerely thank you for your time and interest in taking part of this meeting.

Omar Marianito Maningo Rodis

# Program

| | |
|---|---|
| **09:30-10:00** | **Registration**<br>- Naoyuki Kariya, DDS, PhD |
| **10:00-10:10** | **Welcome Address**<br>- Michiko Nishimura, DDS, PhD |
| **10:10-11:10** | **Development of EMP Education in Japan: Past, Present and Future**<br>- J. Patrick Barron (Professor & Chair, DIMC) |
| **11:15-11:45** | **Development of an EMP core curriculum at Tokyo Medical University: Making it Relevant**<br>- Chieri Noda, MA (Senior Lecturer, DIMC) |
| **11:45-12:15** | **Development of EMP curriculum at Tokyo Medical University: Incorporating the Medical Interview Component**<br>- Aya Watanabe, MA (Research Associate, DIMC) |
| **12:15-13:15** | **Luncheon Seminar**<br>**Communicating Medical Research Results in English Publications Facilitated by Technical Editing & Writing**<br>- Edward Barroga, DVM, PhD (Associate Professor and Senior Editor, DIMC) |
| **13:15-14:45** | **Open Forum** |
| **14:45-17:30** | **Workshop: Drafting the Proposed Core Curriculum**<br>- Participants with Speakers (as advisers)<br>**Afternoon Refreshments** |
| **17:30-17:50** | **Other Matters**<br>- Omar M.M. Rodis, DMD, PhD |
| **17:50-18:00** | **Closing Remarks**<br>- Toshiko Yoshida, MA, PhD |

**THE 1st INTERIM REPORT ON**

**DEVELOPING A CORE CURRICULUM FOR DENTAL ENGLISH EDUCATION**

Submitted to the Ministry of Education, Culture, Sports, Science and Technology
as a progress update report of Grant No. 23531201
for Scientific Research (C) (General) (2011-2014) entitled
Developing a Core Curriculum for the Dental English Course in all dental schools in Japan
日本の大学歯学部（歯科大学）での歯科英語教育におけるコアカリキュラムの開発
「基盤研究（C）（一般）（H23～H25）」

**-Omar Marianito Maningo Rodis-**
2011

## The 1st Discussion Forum to Develop a Core Curriculum for Dental English Education

日本の歯科大学における歯科英語教育コアカリキュラム開発に関するディスカッションフォーラム（第 **1** 回）

Okayama International Center, Okayama City, Japan

August 28, 2011    9:00 AM – 5:00 PM

### Participating universities and representatives

| | |
|---|---|
| Hokkaido University 北海道大学 | Taro Arima |
| Health Sciences University of Hokkaido 北海道医療大学 | Yujiro Handa |
| Iwate Medical University 岩手医科大学 | Yoshinori Sahara / James Hobbs |
| Tokyo Medical & Dental University 東京医科歯科大学 | Naoko Seki |
| Tokyo Dental College 東京歯科大学 | Yoshiaki Shibaie |
| Nippon Dental University Tokyo 日本歯科大学生命 | Chie Yanai |
| Showa University 昭和大学 | Masaki Ohno |
| Kanagawa Dental University 神奈川歯科大学 | Martin Peters |
| Niigata University 新潟大学 | Roxana Stegaroiu |
| Nippon Dental University Niigata 日本歯科大学新潟生命 | Ikuo Kageyama |
| Asahi University 朝日大学 | Hironori Tsuchiya |
| Aichi Gakuin University 愛知学院大学 | Kazuyoshi Suzuki |
| Osaka Dental University 大阪歯科大学 | Junichi Fujita |
| Okayama University 岡山大学 | Omar Rodis / Seishi Matsumura<br>Naoyuki Kariya / Toshiko Yoshida |
| Hiroshima University 広島大学 | Hiroko Oka |
| Kyushu University 九州大学 | Jane Harland |
| Nagasaki University 長崎大学 | Etsuko Watanabe |

## Aims of the discussion forum

1. To initiate a gathering of teachers of Dental English courses from Japan’s 29 dental schools
2. To discuss how to develop and implement a core curriculum for the course
3. To organize a support group for teachers of the course

## Proposed Agenda

Morning Session:

1. Background and summary of current status of Dental English among Japan’s dental schools and student’s competency. (Rodis)
2. Short comments and discussion on syllabi implementation, teaching/learning methods (Participants)

Afternoon Session:

1. Strategies of respective teachers on applying English medico-dental terminologies to the undergraduate dental course.
2. Agreement on "GIO (General Instructional Objectives) and SBOs (Specific Behavioral Objectives)" of at least 2 or 3 years’ courses.
3. Feasibility of offering the course as a compulsory dental subject.
4. Possibility of offering the course for 6 years, or if not, deciding the most feasible.
5. The feasibility of including Dental English in the National Dental Board Exams.

## Minutes of the presentations in the Morning Session

**Welcome address**: Matsumura

Dr. Seishi Matsumura is associate professor of the Department of Behavioral Pediatric Dentistry and a co-investigator of the current grant. He welcomed the participants to the city of Okayama and thanked them for their time and interest in coming. He also presented a brief introduction of how the grant was developed.

**Introduction and background of grant procurement**: Rodis

I came to Japan in 2000 as a 5-year PhD student through the Japanese-government sponsored Monbusho scholarship, then continued as a research fellow and was offered the assistant professorship faculty position in 2007. The dental school eventually requested me to teach part of the dental English course offered in the second semester to 1st year and 2nd year dental students. Unfortunately, the course had no core or model curriculum to base on so I started teaching the course with a self-developed syllabus. In 2008, the course was only offered in the second semester of the 1st year curriculum possibly due to lack of teachers. It was during these times when I searched for background studies and found out that only a few existed. It was then that I decided to conduct needs assessment surveys to my students for updated information that could help improve the course. The idea of procuring a grant to develop a core curriculum came into mind when a survey I conducted to other dental schools in Japan revealed disparities in teaching the course and the nature of the course itself.

The earliest reference on English education in dental schools in Japan was a survey conducted in 1999 by Zac Morse and Sen Nakahara of Nippon Dental University. They reported the lack of consensus among dental schools in teaching dental English courses and concluded that their study has both local and global dental education implications and that this is an area of dentistry that has received little research attention and needs to be reviewed. However, more than 10 years have passed and there is still no core curriculum for the course. It is not even offered in some of Japan's 29 dental schools. An electronic database search for English publications using the key

word “medical English” through Ichushi Web, Science Direct and PubMed conducted in June 2011 revealed 138, 257, and 44 hits respectively while the key word “dental English” revealed 3, 0, and 0 hits respectively. This clearly confirms the lack of studies concerning dental English.

Academic globalization and the directive from Japan’s Ministry of Education, Culture, Sports, Science and Technology (MEXT) called for a shift from mastering grammar toward an emphasis on functional, communication-oriented teaching and the development of a student’s listening and speaking skills and to improve the quality of English education in Japan and produce citizens who can function effectively and be competitive in global society. This led to higher education reforms among universities aiming to produce unique and marketable education programs locally and internationally. In the case of dentistry in Japan, some schools started including dental English courses into their curriculum at their own discretion.

In Okayama University for instance, the course was originally offered for two semesters (second semester of the $1^{st}$ and $2^{nd}$ year) and then revised it to just one semester (second semester of the $1^{st}$ year) after 2008. When the request for me to handle the course came in 2007, I immediately felt the need to develop the course based on the inadequate resources available and the importance of the course in these changing times. Since it was difficult for me to determine how to develop the course, the thought of conducting surveys to my students to assess their current needs came into mind. Surveys were conducted every year and results were consistent concerning the students’ need for more units of the course (see the following figure).

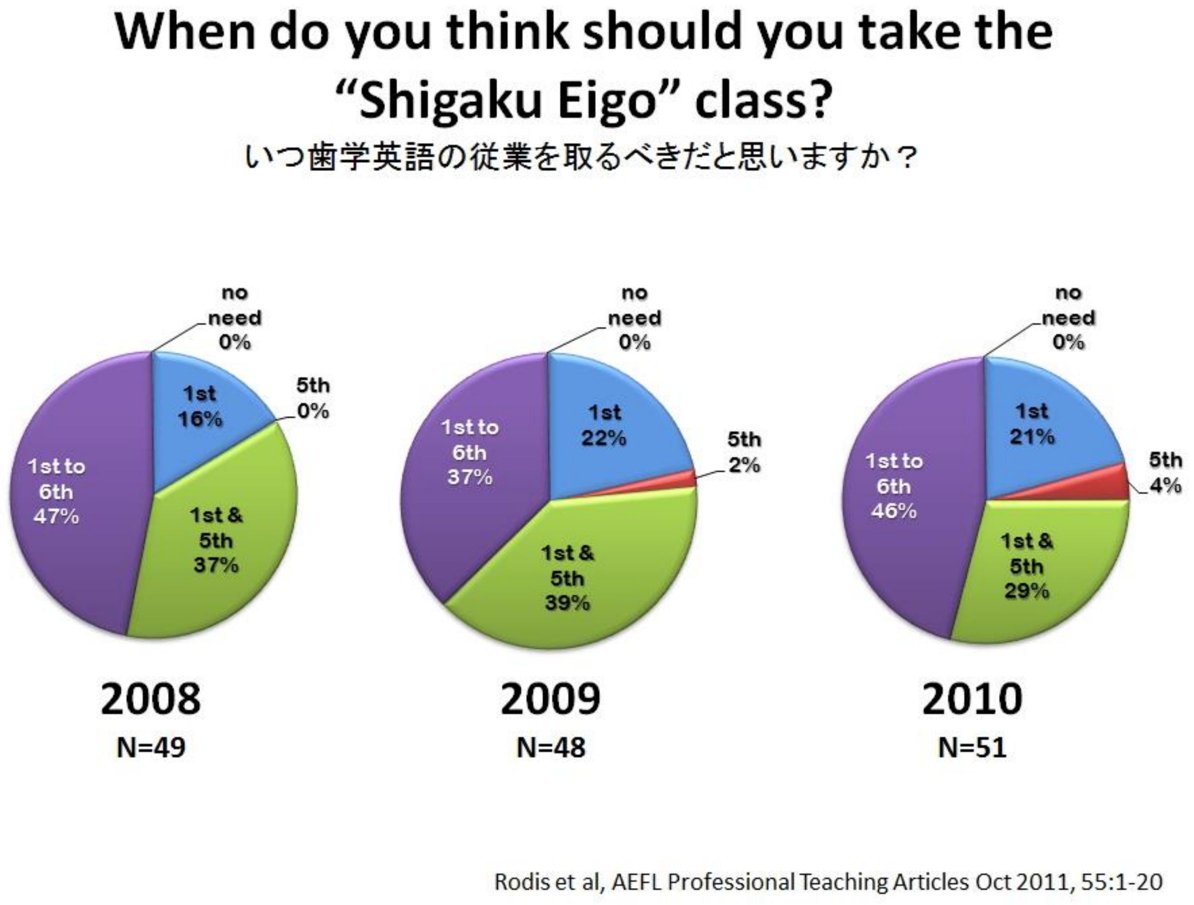


The figure above only shows one school (Okayama University) and it may or may not represent the opinion/s of students in other dental schools. However, it was through this information, the call for globalization, and the lack of background studies that I decided to apply for the grant. The needs of students are constantly changing and with it come the need for schools to regularly assess it as well. There is therefore a need to develop a core curriculum for dental English courses (Dental English, English for Dentistry, English for Dental Medicine, *Eigo Shigakubu*, *Shika Eigo*, *Shigaku Eigo…*). The aim of the morning session for this discussion forum was to gather information through reports or presentations of participants on the current status of the course among dental schools offering it.

**Current Status of Dental English**:

**Rodis** (Okayama University)

- Dental English is only offered for one semester in the $1^{st}$ year.
- Survey results showed students preferring to have more of conversation courses
- Most students prefer to have more dental English courses in their dental years

**Handa** (Health Sciences University of Hokkaido)

- Prof. Handa reiterated that teachers need to distinguish between General and Medical/Dental/Life Sciences English courses.
- What we can do mainly is to give students good exposure to terminologies.
- Positive indicators include growing student interest in the course and collaboration between the teaching staff is very favorable.
- Constraints and challenges include: student’s English proficiency is low, terminologies are not actively used in teaching and training, and most teachers complain about the shortage of time.
- The core problems include: student’s incompetency in using technical terms because basic English language skills is not standardized, students are not well-motivated, students exposure to technical terms during their study is not enough, and bilingual terminology (Japanese & English) usage is not promoted in classes or hospital (clinical) training.

  **Summary**:

  1. Produce competent students who can use technical terms.
  2. Further strengthen student’s overall communication skills.
  3. Having 2 languages together (particularly terminology) throughout the faculty and throughout the course, regardless of topic, so that we can promote a bilingual way of thinking to our students and so that they will be able to talk about their own specialties by maximizing the use of terminologies based on their basic English skills.

**Sahara** with **Hobbs** (Iwate Medical University)

- Prof. Sahara stated that the reason why he was asked by the school to handle the course was because he was the only faculty who had the experience of living in the United States for a long time (6 years).
- 1st year: Basic English education – students are divided into small groups and discuss about common topics in daily life.
- 2nd to 4th year: Medical/Dental terminology and communication (Prof. Hobbs) – students are taught the etymology and structure (prefix, root word, suffix) of technical terms, then divided into small groups and discuss about technical terms, or learn using crossword puzzles. Communication using the terms is taught in the 4th year.
- Due to IMU's not so high board exam passing rate, the school decided to allot more time on board exam subjects and practicum.

  **Summary**:

  1. Hire teaching assistants to partner with students.
  2. English may not be that important within the dental curriculum since the main aim of students is to, first and foremost, pass the board exams.
  3. Foreign students coming to Japan take classes in Nihongo and if they want to get a license, they have to take the board exams in Nihongo.

**Seki** (Tokyo Medical and Dental University)

Asst. Prof. Naoko Seki represented Prof. Ikuko Morio, head of the Dental Education Development Section at TMDU.

Current curriculum (1st to 5th year)

1st yr: Liberal Arts course / General English curriculum

- Composed of Reading, listening, writing, speaking to allow students to brush up on the English skills they learned in high school
- The aim is to develop basic competence in English
- English A, B, C, D and E plus e-learning courses

  English A & C: Oral Communication and Listening

  English B & D: Reading and Writing

English E: Presentation Skills

2nd yr: Same as 1st yr plus Dental/Scientific English & Elective Seminars

- English IIA: Speaking and Communication
- English IIB: Reading, Writing and Vocabulary
- English IIC: Choose from 1st semester's "required & elective" courses
- English IID: Choose from 2nd semester's "required & elective" courses

3rd to 5th yr: Elective Seminars

- Students become busy with their clinical courses and practicals and they will have lesser time to study English.

Elective Seminars

- Each unit is composed of 5 classes
- Students can choose 1 unit in the 1st half and 1 unit in the 2nd half
- Includes lectures on study-abroad experiences and collaborations, ALC Net Academy, Medical English, English communication in clinical and international conferences and Basic English writing.
- Discussing licensure exam questions in English as a review and motivating tool for students.
- Public speaking (presentation skills exercise for international conferences)

  Example: Parts of Speech (Introduction, Body, Conclusion) is just like parts of a scientific paper

For 2012, a revision in the curriculum is planned (EBM, EBD…). Dr. Seki also presented some of her results of a 2008 report about "Demands and Importance of English Education":

Q1. Do you want to have an opportunity to speak English? YES (majority)

Q2. Do you use English in your daily life? NO (majority)

Q3. Is it a good idea to have a lesson like this class once a week? YES (majority)

**Shibaie** (Tokyo Dental College)

Prof. Shibaie is an English teacher. First year students take a general English course followed by basic dental English courses during the first semester of the $2^{nd}$ and $3^{rd}$ year of dental school. He reported that $1^{st}$ years are usually not confident in their English speaking and listening skills.

$2^{nd}$ year GIO – to learn at least 400 basic dental English words with the focus on sound (pronunciation).

$2^{nd}$ year SBO – Acquire listening comprehension using the 400 words

Acquire the ability to pronounce the 400 words correctly

Acquire the ability to make sentences using the 400 words

Acquire the ability to say the meaning of the 400 words

Acquire the ability to spell the 400 words correctly (during exams)

$3^{rd}$ year – Pair-teaching

- Basic dental/clinical science (university dentists)
- Prof. Shibaie

Usually done by reading and discussing a reference/paper (participants are students, Prof. Shibaie and other designated dentists who can explain the clinical implications)

**Yanai** (Nippon Dental University – Tokyo)

Prof. Yanai co-teaches Dental English at NDU-Tokyo. It started as a 3-year course ($1^{st}$ to $3^{rd}$ year) but was reduced to 2 years after 2004 to give more importance to board exam courses. It covers basic and oral communication with emphasis on dentistry.

In 2009, the course was named English for Dental Medicine. The $1^{st}$ year course is purely lecture-style by native English speaking teachers due to the large number of students (100+). In the $2^{nd}$ year, a textbook exclusive for the university is used (written by Prof. Yoshitomi). The book focuses on oral communication. The first semester focuses on lecture while the $2^{nd}$ semester focuses on communication. The aim is to give students the opportunity to talk to as many classmates as possible.

In the future, the aim is to further increase the course units and this can only be achieved through a nation-wide effort. Otherwise, it will be useless for teachers of the course to recommend increasing the course units, and the schools say no.

**Peters** (Kanagawa Dental College)

Prof. Peters is an English teacher and teaches Medical and Dental English. As with other private schools, KDC has financial pressures and therefore welcomes a large number of students. Thirteen years ago, the medical English course enrollees totaled 120 students, and then divided into two groups five years ago, then back to one class of 120 in one big classroom two years ago. Peters further added the need to renew the model of education.

Because of the dropping number of incoming dental students (due to the drop in high school graduates), KDC is currently recruiting foreign students (9 from Korea) with the condition that they should be proficient in Japanese. The textbook he is using was published in the 80's so he was happy to receive a complimentary copy of a new dental English book written by Prof. Watanabe of Nagasaki University.

He also added that students become their own teacher (as in active learning) and it is therefore the task (a big task) of the teachers to make students "active".

He also stated his usage of the PopJisho, a Life Science dictionary developed by Kyoto University, to everybody (www.popjisyo.com).

**Harland** (Kyushu University)

Prof. Harland is a lecturer and teaches ESP and EAP. The new dental English curriculum at KU started in 2008 through a grant from MEXT to establish an ESP curriculum with the aim of improving the professional and academic English skills of dental students. About 50% of students at KU go onto PhD studies. There are foreign students in the undergraduate and graduate schools, and English is the means of communication.

The KU Dental English Curriculum

| | EGP | ESP I | ESP II | Hospital |
|---|---|---|---|---|
| **Year** | 1 | 2 3 | 4 | 5 6 |
| | General | Professional | Academic | Clinic |
| **Description** | | Dental terminology | An introduction to dental research and academic presentation skills | No English program but students can consult a teacher |

The entire English program at Kyushu University is tailor-made to fit in with the students' studies in Japanese.

KU ESP team **=** Native speaker **+** Dentists

- Blended learning = face-to-face learning + e-learning
- Key content includes text files (PDF), audio files (MP3), and video files (QT, WMV) which can be accessed through Blackboard (www.blackboard.com)

**Stegaroiu** (Niigata University)

Associate Professor Stegaroiu presented "Dental English Education at Niigata University". The Dental English course started in 2003 and focuses on chair-side communication in English.

EGP is offered twice a week in the 1st and 2nd semester of the 1st year and the 1st semester of the 2nd year. DE is offered in both semesters of the 3rd year (40 students) and in the 2nd semester of the 4th year (45 students). It is important to know who we are teaching and who is teaching.

Even if the DE teacher is a non-native English speaker, he/she can always teach by motivating students or make them use their English knowledge. Setting herself as an example, she tells her students of her own experience of studying English.

The dental school facilitated the use of teaching assistants (TA) to assist in the teaching of DE. For each lesson, there are 4 TA's in the 3rd year and 3 TA's in the 4th year. The TA's are foreign postgraduate students who are fluent in English.

Regarding the opinion of a previous speaker that DE should be taught throughout the dental curriculum, she stated that it will be impossible for one teacher to teach DE alone up to the 6th year of dental school. In the event that DE teaching is to be extended to all

years, financial resources would be needed to provide additional teaching staff to help in teaching. Thus, the proposed length of the DE teaching has to be adjusted to existing resources.

English Communication Syllabus 1 (3rd yr, 1st sem) focuses on intercultural differences between Japanese and westerners to enhance international communication.

GIO's: The students are expected to grasp the meaning of short essays of a general topic without translating it word for word.

SBO's: The students will be able to answer questions appropriately about the content of the essay and be able to ask questions to fellow students in English as well (student-student interaction).

There are 14 classes per semester, a final examination, and a spare class (for re-examination).

Student assessment is 85% final exams and 15% participation.

Lesson Structure:

Short lessons – listening exercise video/audio and then asking students questions about it

Vocabulary – terms used in the video/audio lessons

Subtitle – listening to audio recordings of the essay to be read and discussed that day

Q&A – example TRUE or FALSE questions

Groupings – Dividing students into groups assisted by TA's

Reading – read lessons paragraph by paragraph with the TA asking questions and discussing answers in English.

TA manual (to provide standardized guides TA's)

Give precise instruction for each lesson

Give ideas on how they have to relate with students

Give list of vocabulary to use

Give list of possible questions about each topic

Give TA's the ability to freely ask questions to further improve student interaction

English Communication Syllabus 2 (3rd yr, 2nd sem) focuses on more complex essays on subjects like science, psychology, etc. without the use of word for word translation.

GIO’s, SBO’s and lesson structure are the same as syllabus 1.

The ultimate aim is to allow students to express their opinions in English.

Dental Chair-side Communication in English Syllabus (4th Year)

GIO: students will learn how to communicate with English-speaking patients on basic topics of routine dental consultation and treatment.

SBO: students will be able to understand what the patient communicates in English and will be able to ask their patients about their medical and dental conditions as well as explain dental procedures in English.

Lesson structure:

Explain dental terminology and phrases commonly used with patients

Listening exercise

Keep lessons short

Role playing (done with TA): Start by reading the dialogue

Practice with TA/partner using the dialogue (similar dialogue is ok)

If too difficult, students are allowed to refer to the Japanese dialogue

Curriculum evaluation:

Students prefer the role play classes (real situations).

The time of the students is an important factor to consider when offering the course in the dental curriculum. Some students may want to have more dental English classes in the senior years but majority of them cannot attend it due to clinical duties (even if they want to).

Offer academic writing and reading after graduation (postgrads).

The core curriculum should be basic for everyone (teachers of the course) to build up. Making the core too big will make it difficult for everyone to reach the specific needs of students.

**Kageyama** (Nippon Dental University – Niigata)

Prof. Kageyama teaches anatomy to medical students at NDU-Niigata.

He had the experience of studying in Germany (2 years), Canada (1.5 years) and Sri Lanka.

English reading comprehension at NDU-Niigata is taught during the $1^{st}$ and $2^{nd}$ year.

As it is with his observations, he shared a report about the 3 S's of Japanese students studying English…

1. S = Silent
2. S = Smile
3. S = Sleeping

Pointed out the importance of learning and using practical English and shared his own learning experience: "is this a pen?"

Stated his reasons why Japanese dental students have difficulty using English (even if they studied English already):

1. All dental textbooks are written in Japanese.
   Dental textbooks used in Sri Lanka, India, Philippines, etc. are all in English
2. National dental board exams are given out only in Japanese so students don't study English.
3. Faculty and staff at dental schools in Japan do not use English as a practical or casual sense of teaching (translating word for word is not good).

**Tsuchiya** (Asahi University)

Prof. Tsuchiya presented "The current state of English education in Asahi University". The dental English course at Asahi University aims to teach English for Dental Purposes as a tool for studying Science, Odontology and the NDLE (National Dental Licensing Examinations). He started teaching the course about 10 years ago.

He also spoke about the need to update the curriculum at Asahi University to address the changes proposed by different medical, dental, educational societies concerning the inclusion of English in the exams of the professions.

Asahi University's immediate aim is to develop dental English education as soon as possible. The school is also aiming to improve the passing rate of their students for the National Dental Board Exams in line with the inclusion of English in the said tests. It is also important to constantly motivate students into learning English. It is also important to provide students with a clear outline in learning English.

**Oka** (Hiroshima University)

Hiroshima University offers dental English courses (1 and 2) as an elective course for 3rd year dental students in the first semester. Course 1 focuses on experiences of postgraduates living abroad. Course 2 focuses on discussion lessons on dental research topics. For the 4th year, the course focuses on bedside/chair-side courses and research. Chair-side courses are prepared for all 4th year students and divided into levels of English comprehension. For top level students, chair-side conversation, research, thesis-writing and training for international conference presentation are taught while for low level students, common daily conversation is taught. Additionally, students of Frontier Dental Sciences course join into research. Their research lectures include reading and discussion of scientific papers.

From October 2012, Hiroshima University will start a new course called International Dental English course. In 2013, the course will be re-evaluated for the possibility of offering it from 2nd to 5th year. The course guidelines are currently being developed by Hiroshima University.

**Watanabe** (Nagasaki University)

Dr. Watanabe and her husband (Prof. Ikuya Watanabe) worked at a dental university in Texas, USA. They are now teaching students at NU about their personal experience in the US and how they learned English. The textbook used for Dental English courses at Nagasaki University was written by multiple faculties of NU School of Dentistry, including Prof. Watanabe, and focuses on clinical and dental conversation (speaking and listening). It is currently used by their 4th year students.

Until 2010, reading and writing was offered to 1st year second semester students. However, starting next year, DE will be offered to 1st and 4th year students focusing on

conversations in dentistry based on a revised version of their university book. Also, NU School of Dentistry proposes that taking the TOEIC test at the beginning of the 1st year will be mandatory and another TOEIC test at the 4th year will be a requirement for DE course starting next year.

## Summary of the discussions in the Afternoon Session

The purpose of the afternoon session was to conduct an open forum about the course curriculum, its objectives, needs, advantages and disadvantages. The agenda of the afternoon session was supposed to discuss 10 items but was revised by the participants on the day of the meeting due to overlapping of issues and to save time. The final agenda were: 1] Strategies of respective teachers on applying English medico-dental terminologies to the undergraduate dental course; 2] Agreement on "GIO (General Instructional Objectives) and SBOs (Specific Behavioral Objectives)" of at least 2 or 3 years' courses; 3] Feasibility of offering the course as a compulsory dental subject; 4] Possibility of offering the course for 6 years, or if not, deciding the most feasible; and 5] The feasibility of including Dental English in the National Dental Board Exams. Since this was the first ever discussion forum on dental English courses, it was inevitable that the discussions would be a free discussion allowing every participant to express his/her own experience and opinion.

The discussion forum, albeit presented positive and negative opinions on a wide variety of issues, was useful in presenting the real problems (and some solutions) faced by teachers of dental English courses unknown previously to most of the other participants. Due to the applicability and similarity of opinions on each of the five items, they were further grouped into A] Primary Objectives and Teaching Strategies (Items 1 and 2) and B] Future Objectives (Items 3, 4 and 5) in this report.

A. Primary Objectives and Teaching Strategies

In developing the core curriculum for dental English courses, it is important to note that the primary objectives are to teach and motivate future Japanese dentists to be

confident in speaking English to foreign patients or international colleagues, to know common medico-dental terminologies, to be confident in providing dental treatment to all patients including foreign patients, and to be ready to improve their professional and career paths by also thinking global and not just local. The core curriculum should also be developed with a consensus and in a way that should be readily adaptable to new and existing curricula of the different dental schools in Japan. For the current grant, the objective is to facilitate a fact-finding opportunity, summarize these facts and present a recommendation to MEXT.

Teaching strategies were discussed based on the following issues: motivation, medico-dental terminology and patient/inter-profession communication.

*Motivation: Motivating students to learn is a big issue to consider in curriculum development.*

- developing programs to motivate students at a certain period in their dental years.

- offering many types of programs for students to find their "own way" of studying English.

- offering exchange programs (both abroad and within Japan).

- increasing their awareness of local and global competitiveness.

- creating a "culture" within the dental faculty and dental students to use dual language.

- providing alternative/additional learning opportunities to students that should fit around their time/lifestyle. Having alternative learning materials can motivate students by allowing them to preview upcoming topics or review past lessons. This includes internet/web-based learning materials such as Blackboard and Moodle (www.moodle.org).

- providing a word-list of commonly used English (general, dentistry, medicine).

- providing special listening or speaking equipment.

- using these terms in context for conversational purposes (and not just teaching the term).

- making classes interactive. Students should be walking around, asking questions, or conversing with the teacher or other students.

- emphasizing to students the duty of the dentist which is to provide dental care and treatment to everyone (Japanese and foreign patients).

- offering regular graduate programs run in English is an advantage to students when they write or present their reports or thesis.

- offering continuous dental English courses throughout the dental years to provide a refresher for students.

- assessing TOEIC/TOEFL/EIKEN scores to allow students/teachers to choose the proper level of learning/teaching because general English is the foundation for professional English.

- reiterating to students the importance of English as a common and practical second language even if they choose to work in Japan.

*<u>Medico-dental terminology</u>: There is a big gap between language instruction and content instruction wherein one extreme is teaching English language topics and the other extreme is teaching dental topics in English.*

- offering courses in Latin, Greek, English and Japanese (for example in Oral Anatomy class).

- implement bilingual teaching by teaching staff.

- develop teaching (PowerPoint) slides that show Japanese and English terms with listening or pronunciation exercises.

- using “blended learning” in teaching the courses. If human resources are adequate, blended learning is known to be effective in teaching English for professional/academic purposes. It utilizes the teaching efficiency of the English

teacher, the teacher of the profession (dentist, doctor…) and the teaching materials (multi-media, internet, web-based…).

- having shorter class periods can help students retain the subject matter (45 minutes).
- having a short break between classes (in case of longer class hours).
- length of classes will also depend on the level of proficiency of the student (learning a second language for long hours is difficult for a beginner).
- providing students a list of basic terminologies of basic science (anatomy, biology, physiology…) or those listed by “monkasho yo gou”.
- incorporating the word-list into the core.
- using books that have both English and Japanese terms and dental English for conversation.
- providing balance between basic and technical knowledge.
- offering a course for basic/easy terminology and technical terminology.
- mixing basic conversation and dental conversation to allow students to use the terms in a sentence and be able to understand/remember the term more.
- teaching the terms in context. Teachers should explain first the category (general overview) of the new term then explain the details next.
- allowing students to learn the terminology with its English meaning, pronunciation and giving students something communicative to do.
- incorporating terms with composition courses (translation with context). For example asking students “how do you say, I want to go to the Pediatric Dentistry department?” while also giving them the terms of other departments so they can use the terms accordingly.

*Patient communication and inter-profession communication: Dental English courses should also be able to prepare future Japanese dentists to communicate confidently in English with foreign patients and foreign colleagues.*

- teaching common terminologies and abbreviations used in different international settings. For example, premolars are sometimes referred to as bicuspids in the USA; or the use of the ITDS (International Two-Digit System) in tooth nomenclature in the USA.

- basing the core curriculum with what is happening in reality... students must be able to relate general English with dental English (in conversations with other professionals or patients).

- emphasizing the importance of becoming global dentists.

- financial and human resources are important in implementing a comprehensive curriculum.

- offering extra-curricular activities for visiting scholars and students to allow students to speak with them.

- teaching students alternative and simple conversational phrases for patient communication (even if incomplete) like for example; "which tooth?" in case they forget the complete sentence.

- providing opportunities for students to act as campus tour guides for foreign visitors.

- providing more oral conversation opportunities during the undergraduate English for general purposes classes.

- providing extra-curricular activities for students to be able to interact with foreign students or opportunities to participate in study-abroad programs.

- providing special seminars on English oral presentations, thesis-writing or journal clubs during the senior years.

. Future Objectives

The afternoon session also discussed on future objectives including the inclusion of dental English in the national dental licensure examinations. Below is a summary of the issues related to the future objectives raised during the discussion forum:

- the possibility of integrating dental English courses into the OSCE (Objective Structures Clinical Examination) by introducing a question (or case study) in English. This could be one way of motivating students to study English.

- integrating dental English into OSCE may cause difficulties or too much burden to students.

- integrating into OSCE may also cause problems on those who will implement it or those who will develop the test.

- practicing English conversation is important for dental students and the OSCE could be a good learning experience for them. Thus, to address the above-mentioned problems, it is possible to just have the questions (or the station) as optional or ungraded.

- even if questions/stations are optional or ungraded, integrating into OSCE could still pose a problem to students who may not have good English-speaking skills.

- the possibility of integrating dental English courses into the national dental licensure examinations (similar to that of the medical board exams). This could be beneficial for students as a means of a second assessment of their English-speaking skills since there are some students who may have high TOEIC scores upon entering dental school but are unable to communicate well after dental school due to lack of continuing education opportunities.

- integrating dental English courses into the national dental licensure exams may affect the continually decreasing passing rate for the licensure exams and the possibility of the reduction of the number of dental schools in Japan in the future.

- the concern that there may be limited job opportunities for future Japanese dentists due to international laws concerning the practice of dentistry.

- a suggestion to focus on developing the core curriculum first instead of the board exams was expressed since it is the main purpose and is also the first step for a successful meeting.

- ASEAN countries are improving their English skills and the possibility of Japan being left out or marginalized is imminent if English is not taught throughout the learning years.

- Associations for medical education in Japan have been improving the English education of medical students so this should also be the case for dentistry.

Some of the participants thought that integrating dental English courses into didactic, clinical exercises (OSCE) or national dental licensure exams might be a way to motivate students to study English seriously while others voiced out their reservations as to its implications to the overall passing rate of examinees in the licensure exams. Nonetheless, Japan's leading role in the academics, research science and technology in the Asian region could be compromised if English education is not improved and implemented systematically. Although no conclusions on GIO's and SBO's were reached, the participants decided to leave the issue for future meetings and to concentrate instead on developing the core curriculum first.

## Other Matters

Other important matters were discussed at the end of the meeting. This included plans for the 2nd meeting, MEXT representation and a support group or society.

*Plans for the 2nd meeting*

The 2nd meeting will be held around May 2012 and will be a workshop/seminar. The exact date and venue will be decided by a consensus (Survey Monkey). The objectives

of the meeting will be to develop the core curriculum based on dentist-patient communication and inter-profession communication and developing the GIO's and SBO's of the course. Participants of the meeting will be the same and if financial and human resources permit, participants from unrepresented schools will also be sent invitations to participate. A report on the minutes and summary of the 1st meeting will also be compiled and sent to all participants.

*MEXT representation*

It was agreed that a draft for the core curriculum should be developed first before inviting representative/s from MEXT. The 2nd meeting will be a good opportunity for a consensus on what should comprise the core curriculum.

*Support group*

For the benefit of teachers and future teachers of the dental English courses, it was discussed if a support group should be organized. It was decided that since the group (and the course) is still new, joining an existing organization in a related field would be best. The Japan Society for Medical English Education is open for membership in the paramedical fields and this option will be discussed in detail during the 2nd meeting.

**Closing Remarks** (Yoshida)

Prof. Yoshida is an assistant professor at the Center of the Development of Medical and Healthcare Education (Dental Education), Okayama University. She thanked everyone for attending the meeting and hopes that the discussions will be beneficial for developing a simple core curriculum for dental English courses and for the dentistry in Japan as a whole and wishes everyone a safe trip home.

## Interim Conclusions and Recommendations

The 1st discussion forum proved to be a successful gathering of educators responsible for dental English education in their respective schools. The presentations of the current status of English courses in dentistry from each school and the fact-finding discussions proved to be a good source of base knowledge for the development of a core curriculum for these courses. The disparity between and among schools also reiterates the need for school and education officials to address it systematically. Although the discussions covered a variety of issues, most of it focused on the importance of motivation and dental English terminology as basic requirements in preparing students to communicate confidently in English with international patients and colleagues. The presentations of participants on how this was implemented in their respective schools (including problems they have encountered and what they did to overcome it) provided insights to other participants as well. Although only 17 out of Japan's 29 dental schools were in attendance, we hope that the 2nd meeting will see representatives from all schools.

A workshop seminar is planned for the 2nd meeting to be held possibly in June 2012. The workshop will focus on formulating the GIO's and SBO's of the core curriculum involving patient-dentist communication and inter-profession communication. The seminar will have speakers from the Japan Society for Medical English Education (JASMEE) or other related organizations talking about topics concerning the development of medical English courses and its past and present problems. JASMEE provides a forum for the exchange of new ideas and techniques dedicated to English teaching and learning within the medical/paramedical field.

There was also interest on having a support group for dental English teachers. It was discussed that it might be beneficial to join JASMEE since dental English education is still new. JASMEE organizes conferences on medical English education in Japan annually.

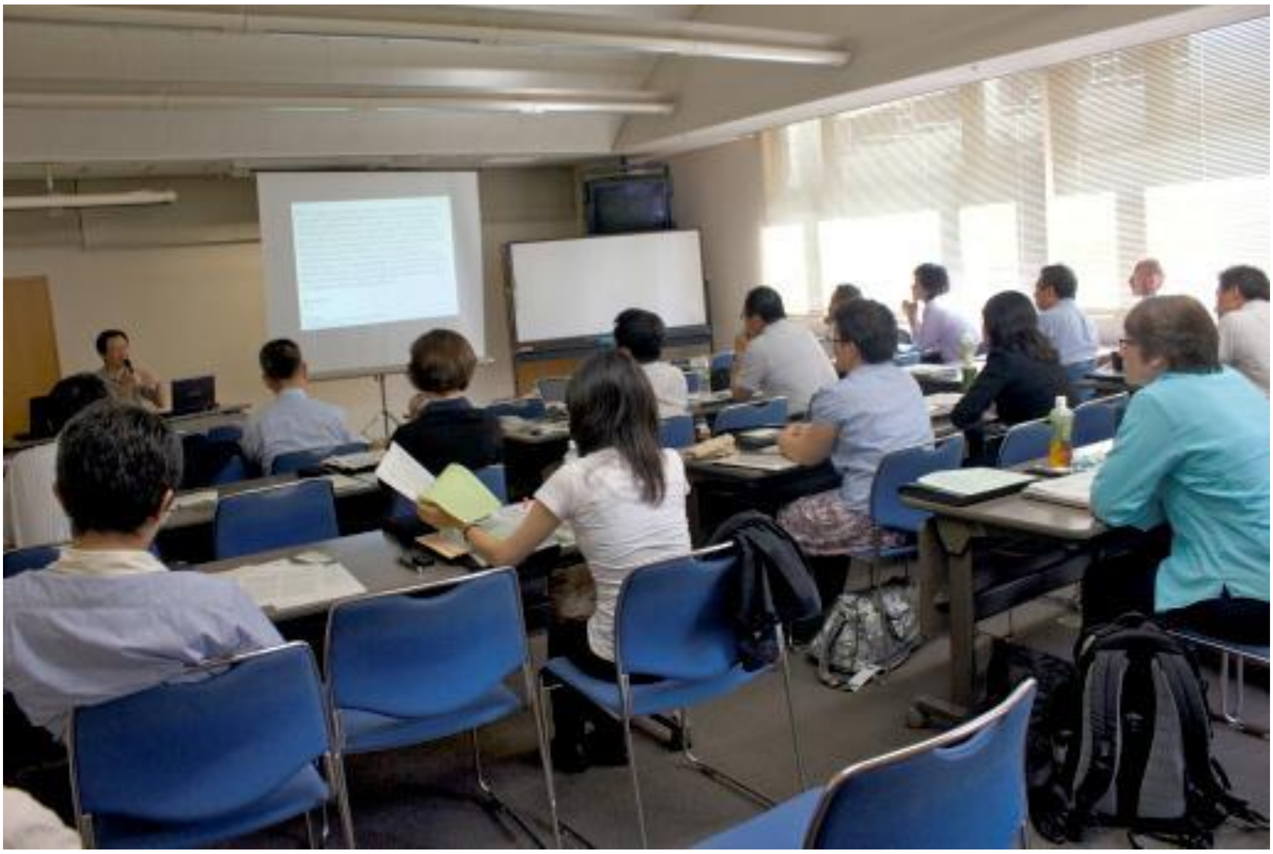

On behalf of my co-investigators, I would like to extend my sincerest thanks and appreciation to the participants of the 1st discussion forum. Your presence, interest and invaluable comments and suggestions made our meeting successful. I hope to see you again in our future meetings.

Omar M.M. Rodis

# THE 2nd INTERIM REPORT ON

# DEVELOPING A CORE CURRICULUM FOR DENTAL ENGLISH EDUCATION

Submitted to the Ministry of Education, Culture, Sports, Science and Technology
as a progress update report of Grant No. 23531201
for Scientific Research (C) (General) (2011-2014) entitled
Developing a Core Curriculum for the Dental English Course in all dental schools in Japan
日本の大学歯学部（歯科大学）での歯科英語教育におけるコアカリキュラムの開発
「基盤研究（C）（一般）（H23～H25）」

**-Omar Marianito Maningo Rodis-**
2012

## The 2nd Discussion Forum to Develop a Core Curriculum for Dental English Education

日本の歯科大学における歯科英語教育コアカリキュラム開発に関するディスカッションフォーラム（第２回）

Tokyo Medical and Dental University, Tokyo, Japan

June 9, 2012　10:00 AM – 6:00 PM

## Participating universities and representatives

| | |
|---|---|
| Hokkaido University 北海道大学 | Shigeru Takahashi |
| Health Sciences University of Hokkaido 北海道医療大学 | Yujiro Handa |
| Iwate Medical University 岩手医科大学 | Yoshinori Sahara / James Hobbs |
| Ohu University 奥羽大学 | Naomi Fukai |
| Nihon University at Matsudo 日本大学松戸 | Tomiko Yamagami |
| Tokyo Medical & Dental University 東京医科歯科大学 | Naoko Seki |
| Nihon University School of Dentistry 日本大学歯学部 | Clive Langham |
| Showa University 昭和大学 | Masaki Ohno / Yo Shibata |
| Kanagawa Dental University 神奈川歯科大学 | Martin Peters |
| Tsurumi University 鶴見大学 | Asiri Jayawardena |
| Nippon Dental University Niigata 日本歯科大学新潟生命 | Ikuo Kageyama |
| Asahi University 朝日大学 | Hironori Tsuchiya |
| Aichi Gakuin University 愛知学院大学 | Kazuyoshi Suzuki |
| Osaka Dental University 大阪歯科大学 | Junichi Fujita |
| Okayama University 岡山大学 | Omar Rodis / Michiko Nishimura<br>Naoyuki Kariya / Toshiko Yoshida |
| Hiroshima University 広島大学 | Hiroko Oka |
| Kyushu University 九州大学 | Jane Harland |
| Nagasaki University 長崎大学 | Etsuko Watanabe |

## GUEST SPEAKERS

**J. Patrick Barron**　バロン・パトリック
Professor and Chair
Department of International Medical Communications
Tokyo Medical University

**Edward Barroga**　バロガ・エドワード
Associate Professor and Senior Editor
Department of International Medical Communications
Tokyo Medical University

**Chieri Noda**　野田 千ゑ里
Senior Lecturer
Department of International Medical Communications
Tokyo Medical University

**Aya Watanabe**　渡邉　綾
Research Associate
Department of International Medical Communications
Tokyo Medical University

## AIMS OF THE 2$^{nd}$ MEETING

**1.** To organize a workshop/seminar.
**2.** To gain knowledge and advice on core curriculum development from invited speakers.
**3.** To discuss and decide the contents of core curriculum.

## THE SEMINAR

### Development of EMP Education in Japan: Past, Present and Future

The first speaker was Professor J. Patrick Barron, who shared his experience in developing medical English education in Japan 40 years ago and his outlook. Born in Scotland, he graduated from the University of Pennsylvania and went to study Japanese in Japan in 1969. He began his career in medical writing in 1970 under the aegis of Dr. Yoshihiro Hayata of Tokyo Medical University (TMU). He pioneered the field of English for medical purposes in Japan, and is on the editorial board of many journals, including CHEST, the Journal of Bronchology, Respirology, and edits the abstracts of the Japan Respiratory Society journal, as well as the Japanese Journal of Gastroenterology and the Japanese Journal of Gastrointestinal Surgery. He has translated from Japanese or edited about 15 medical textbooks, two of which have won awards. In 1991, he established the first medical communications center in Asia devoted to improving the flow of medical information from Japan to the International medical community, at TMU, providing support to staff submitting and resubmitting papers. Publications from TMU have increased 700% in the past 20 years. As Vice Chairman of the Japan Society for Medical English Education, he provided the initial impetus for the first medical testing of English competence for medical professionals in Japan, which began in 2007. In July 2009, the Department of International Medical Communications (DIMC) was established at TMU, and Professor Barron was elected as the chairman of the department.

In 1982, Professor Barron conducted a survey on the feasibility of developing medical English education in Japan to 430 individuals and school officials in more than 80 medical schools. There were only 48 questionnaires returned from 32 schools. In the end, only 4 individuals, including him, managed to meet and discuss. At present, there are about 600 members and 80 schools who are now members of the Japanese Society for Medical English Education (JASMEE). Professor Barron emphasized the similarity of problems between dental English education at present and at the time when he started developing medical English education in 1982. This included the

length and number of classes offered, teaching staff, communication facilities, teaching materials, and course content. Subsequent meetings addressed most of the problems and medical English courses were eventually offered in medical schools nationwide. Awareness of the program through the surveys and meetings proved to be an important tool in the improvement of the stated problems. Professor Barron further stated that future needs for medical English education should include:

1. Construction of effective curriculum of medical English (early exposure), balanced with conversation and writing, developed based on consultation with basic and clinical medical specialists.
2. Development of texts, designed specifically for such a curriculum.
3. Development and training of teachers capable of working with such a curriculum and texts.
4. Smaller classes, if possible at least 10.
5. Better communication between language teachers and basic and clinical specialists.
6. Participation of language teachers in the communications of the medical school (Medical School Communications Center)

From 1991 to 1993, English for Medical Purposes (EMP) at Tokyo Medical University significantly changed with the establishment of the International Medical Communications Center (IMCC), the first of its kind in Japan. Since then, the university began teaching EMP to 3$^{rd}$ year medical students and further offered it to 1$^{st}$ year students as well in 2003. In 2005, EMP was embedded in clinical organ-based medical education program for 3$^{rd}$ and 4$^{th}$ year students. This allowed students, language teachers and clinical medical specialist to interact and consult each other during English communication laboratory classes through video monitors. At present, the university is offering departmental education in medical career construction for postgraduates. Some important topics include:

1. Uniform requirements for biomedical manuscripts
2. Getting published by top journals
3. Rules that must be observed to increase acceptance

4. Oral presentations and handling questions
5. Plagiarism and copyright

Eventually, the IMCC became a department and is now known as the Department of International Medical Communications. It has expanded its scope by offering personalized medical editing services. This system has increased the research publication output of the university to 700% in 2011 based on outputs from 1989. Professor Barron reiterated the need for international medical communication centers to be established based on current events. He believes that international societies based in the Asian region will increase in number. This has started with the increase of international medical societies with headquarters in Japan, Asian countries, and China, where the increase has been remarkable in recent years.

At the same time, JASMEE continued to produce educational materials and train staff who could improve medical English education in Japan. The society also continues to organize annual meetings, seminars and workshops to address perennial and potential problems, share new teaching and learning techniques or materials, and publish books for both teachers and students. Currently, a 3-book series on medical English is available for course reference. Volume 1 is Building Vocabulary and Reading Comprehension by Masako Shimizu, Volume 2 is Entering Scientific English in Context by Neil Kennedy, while Volume 3 is Principles and Practice of English for Medical Communications by J. Patrick Barron. Professor Barron also developed a webpage for public access called Ronbun.jp Manuscript Primer [http://ronbun.jp/]. The webpage allows users to access information on how to write and publish good articles, advices on how to respond to reviewers, and how to write cover letters among other services. Professor Barron further stated that the urgent needs of medical English education in Japan are: establishing centers that will facilitate the flow of information to the international audience, an improved English education in medical schools, and the use of common educational texts for use in medical schools throughout Asia. He also noted some limitations including the huge need of medical language specialists, educators and translators to staff centers and to facilitate medical communications, the fact that the burden on clinicians and basic preclinical

scientists at present is too great, and the lack of financial resources to establish communication centers and support its staff. However, he reiterated the need for a common and united approach concerning medical English in non-English speaking countries.

Professor Barron's take home message to the participants were (quoted):

1. I praise Dr. Omar Rodis for being able to do what I tried to do, and failed, in 1982.
2. A national consensus is essential to development of a reasonable curriculum.
3. Establishment of a communications center is financially effective.
4. The academics who run the center (editing) can also teach (一石二鳥).
5. Ability to acquire latest information internationally is important for LEGAL and FINANCIAL reasons (legal disputes, research funds, career).
6. Be sure to approach JASMEE this month regarding a Dental Chapter.
7. For medical writing, consult www.ronbun.jp

**Development of an EMP core curriculum at Tokyo Medical University: Making it Relevant**

The second speaker was Ms. Chieri Noda, who talked about the "Development of an EMP core curriculum at Tokyo Medical University: Making it Relevant". Ms. Noda is Senior Lecturer at the Department of International Medical Communications at Tokyo Medical University. She has extensive experience in interpreting for various fields ranging from the law court to Disney Sea. Since 2004, she has been engaged in various projects in the field of medicine. This has included giving English language assistance to Japanese medical researchers and coordinating linguistic validation projects on quality of life questionnaires. She has taught English at both commercial language schools and Japanese medical schools. Just before joining Tokyo Medical University, she wrote her master's thesis for Birkbeck, University of London, on the communicative strategies used by medical researchers in the UK and Japan. As

senior lecturer at Tokyo Medical University, she is responsible for the 3rd and 4th year Medical English program.

Ms. Noda first mentioned about curriculum planning, specification, program implementation, and class implementation as the developmental stages of EMP. The English ability required of all doctors at TMU is provided by EMP courses on medical Terminology, Doctor-Patient Communication, and Acquisition of Medical Information. Additionally, English ability required for an academic career is provided by EMP courses on Research Papers and Presenting at Conferences. Ms. Noda then presented TMU's 3 Core Teaching Materials through the Gendai GP Grant composed of Clinical Concepts (embedded into the organ system-based clinical curriculum), Selected Readings (skimming and scanning research articles), and Medical Interviews (to be presented by Ms. Watanabe). With clinical concepts, there is a need to closely monitor the synchronization and collaboration with the clinician. Ms. Noda presented how this is done using video monitors between the students and clinical instructors. The clinical instructor stays in a room where video feeds from the classroom are transmitted to pre-assigned monitors. This setup allows students to receive a prompt reply from instructors on questions pertaining to a technical or clinical topic. The communication exchange and the impromptu questions elicited by the students become opportunities to practice conversation in a supervised clinical setting. With selected readings, abstracts and introductions sections of research articles from the New England Journal of Medicine are assigned to students. She states that exposing students to real research articles is a good thing to improve their reading skills in the medical/clinical fields but it should be noted that the students have to be guided through since they have not been exposed to the actual scientific field yet.

There is also a necessity for ongoing needs analysis especially from students. Recently, they had the opportunity to receive feedback from eight of their best 6$^{th}$ year students who went abroad to get clinical experience. The students were very excited about the experience and reiterated the importance of medical English. They even requested that there should be more units of the medical English course and that it should be offered to all students. DIMC is now on the process of developing specific

questions for the student feedback to identify situations or contexts where the students were able to use (or not use) their knowledge on medical English. Common difficulties observed include lack of reading skills and lack of familiarity with the research subject and the journal itself, which could be addressed by incorporating language instruction and a review of the structure of research papers.

**Development of EMP curriculum at Tokyo Medical University: Incorporating the Medical Interview Component**

The third speaker was Ms. Aya Watanabe, who talked about the "Development of EMP curriculum at Tokyo Medical University: Incorporating the Medical Interview Component". Ms. Watanabe works as a Research Associate at the Department of International Medical Communications in Tokyo Medical University. As a bilingual, born and raised in Japan, her area of interest has been in Bilingualism, Second Language Acquisition and English Education. After graduating from the Department of English Language and Studies at Sophia University, she received her Master's degree from the University of Hawaii at Manoa, majoring in Second Language Studies. Her research focused on using Conversation Analysis as a methodology to investigate classroom interaction and language acquisition. She is currently engaged in the field of English for Medical Purposes (EMP) at Tokyo Medical University, assisting with the curriculum development and materials development for third and fourth year medical students.

Ms. Watanabe took up the third concept of their Gendai GP Grant, which is the Medical Interview. It mainly focuses on genuine doctor-patient conversation listening materials. She explained the contents as the following: Setting goals and objectives for educational purpose; using blended learning for educational strategy; using a special EMP website for online video materials; using worksheets for classroom materials; implementation; evaluation; promoting feedback from students and instructors; and drawing future considerations. The general objective is to be able to allow students to conduct medical interviews in English by identifying the structure of

medical interview, producing sets of questions appropriate for medical interview, recognizing key questions asked in primary encounters, describing importance of non-verbal communication, defining what connected speech is, and using appropriate verbs for giving medical instructions. As for educational strategy, they promote blended learning, which is a mix of classroom, online learning, and authentic doctor-patient interaction for their audio-video materials. Online learning is facilitated by logging on to TMU's EMP website which provides reading and video materials for free. The reading materials allow students to improve their skills for reading medical research articles while video materials allow students to acquire conversational skills useful in clinical settings. Students log into the system and choose a clinical setting and actual doctor-patient videos are shown. Students may also opt to have the transcripts shown to guide them through or simply listen to the conversation and answer a questionnaire at the end of each session to assess his/her own level of comprehension of that particular clinical setting. Classroom learning is enhanced by using worksheets or checklist for students to fill out after listening to audio-video clinical scenarios. Different learning techniques such as true or false questions, list of terminology, role playing with transcripts, linguistic aspects among others, are incorporated as a classroom learning material to prepare students for the actual doctor-patient consultation. In implementation, the focus was on connected speech, which is common to all languages (ex. I want to = I wanna). As with other methods of evaluation, the written exam was used. In terms of overall student feedback, Ms. Watanabe reported strong interests among students concerning the learning materials while for overall instructor feedback, it was recognized that the video materials for medical interviews were too difficult, and therefore not suitable for students. It was agreed that a simpler and shorter materials be developed. Finally, Ms. Watanabe presented their future considerations for EMP at TMU where a more focused approach on the medical and linguistic aspects and language use within specific context is envisaged.

**Communicating Medical Research Results in English Publications Facilitated by Technical Editing & Writing**

The fourth speaker was Professor Edward Barroga, who talked about "Communicating Medical Research Results in English Publications Facilitated by Technical Editing & Writing". He obtained his Doctor of Philosophy specializing in surgery and oncology from the Graduate School of Veterinary Medicine, Hokkaido University, Japan, and Doctor of Veterinary Medicine specializing in internal medicine and pathology from the University of the Philippines. In the University of the Philippines, College of Veterinary Medicine, he served as Instructor of the Department of Parasitology and Protozoology, Assistant Professor and Head of the Pathology Laboratory, and Chairman and Associate Professor of the Department of Veterinary Para-clinical Sciences. In Tokyo, Japan, he served as Biomedical Editor of MYU Research and Senior Editor of ThinkSCIENCE, Inc. He is currently Associate Professor and Senior Editor of the Department of International Medical Communications of Tokyo Medical University. Professor Barroga is well published in peer-reviewed scientific and editing journals in the UK, Germany, Japan and the Philippines, and has over 20 years of experience as an academician, medical researcher, author, and medical editor.

Professor Barroga talked about the importance of having scientific writing in the core curriculum as it will enhance patient-doctor communication, inter-profession communication and medical research communication. Materials such as posters, presentations, abstracts, research articles and books will enable students to practice and develop their reading, hearing, and communication skills. Alternatively, it also holistically enhances the culture of science publishing within the institution. He emphasized the importance of an electronic editing service within the institution basing on the hundred-fold increase in publications TMU has had in recent years. Their editing services are personalized (consultation with authors as necessary) and continue until the paper is accepted for publication. Complete services include: Comprehensive editing, Cover letter editing, Interpretation of journal decision letter, Resubmission editing, Editing of responses to reviewers' comments, Rejection and

new submission editing, Poster and slide presentation text editing, Oral presentation script editing/coaching, Assistance with guidelines for authors, Assistance with online submissions, and Galley proofreading. This integrated approach of TMU includes developing a comprehensive and standardized English core curriculum, establishing electronic editorial services, developing academic editors, and encouraging the culture of science publishing. Prof. Barroga ended with a note encouraging the participants to develop a core curriculum for dental English education that will include scientific writing courses.

## MINUTES OF THE OPEN FORUM

The Open Forum paved way for participants to ask questions about the respective topics of the speakers and/or suggestions as to how it relates to the development of the core curriculum or the situation at their respective schools. The four guest speakers took turns answering questions raised by the participants and also provided advice to some concerns. Among the comments, questions and concerns raised, the following were the most discussed:

*Concerning TMU's acceptance rate of papers submitted for publication by the time DIMC was established*:

After the electronic editing services started, an increase in the number of published papers ensued and the department has now become a model for institutions in China and Korea. As an expert on medical editing and publication, Professor Barron and his colleagues offered some advice on manuscript handling and rejection. A manuscript that has been rejected does not necessarily mean it is not publishable anymore. Even if manuscripts are rejected, comments from editors and reviewers should be taken into account to rewrite and improve style and content of your paper. Unfortunately, not many students and researchers in Japan are aware of this and not many know that as scientists, they have a moral obligation to get science out into the world for the benefit

of patients. It is therefore important for them to write manuscripts correctly and be guided all throughout the submission process. Thus, incorporating scientific writing courses into the dental English core curriculum is recommended.

*Concerning the value of medical communications center (DIMC) and how it can be seen as a valuable component medical communications center and in-house editing service to other universities:*

The first concern is the cost/expense. Professor Barron personally feels that DIMC is very much appreciated now compared to 30 years ago. A center should be able to increase the number of publications since it is an important index for university ranking. The cost for maintaining a center is high but the advantage of publishing more papers far outweighs it. Also, more and more universities have now come to a realization of the value of publishing and professional editing system within the university. However, many universities are still outsourcing their papers to commercial editing services. Thus, it will surely benefit universities if each would have dedicated in-house professional editing services.

*Concerning the level of comprehension of students and its relationship with level of motivation:*

For example, there are students who are highly motivated, average and those with no interest at all. The highly motivated groups already have an above-average or high proficiency level of English but are a minority while the average and no interest group are a majority. So a question was raised on how to handle all 3 groups in terms of teaching terminology and English and how to raise the students' level of motivation. The speakers acknowledged that raising the motivation level of students in extremely a challenging task. In terms of teaching terminology, TMU teaches 1$^{st}$ year students the principles of terminology (combining forms, prefixes, suffixes…). In terms of teaching English, the key is repetition. This was evidenced by a group of returning study-abroad students who proposed being tested repeatedly. TMU has their e-learning courses to address it. TMU also investigated segregating students according

to level of comprehension (3 groups for high level and 3 groups for low level students). However, they concluded that the system was not effective because it was observed that some students who had high English comprehension were not highly motivated to learn English further (An example given was of one student who has no plans of working abroad). Thus, TMU returned back to the mixed group and found out that this was more effective because highly motivated students (even if they do not have a high level of English comprehension), were observed to be helping out each other or their classmates, which enhanced learning. Ideally, the perfect class should have equal percentages of well/less motivated and good/bad students. It is also important to give students different ways or varieties to learn throughout the length of the course, and not just sticking to one teaching style.

*Concerning the fact that most students do not see any use of English for their careers because they will practice in Japan and treat Japanese patients, a question was raised on how to effectively motivate them to become globally-competitive*.

The disparity is evident between and among public and private schools. The participant, who asked the question, attended a conference in China and was surprised to see Chinese students confidently speaking and discussing with other delegates in English, in contrast to Japanese students. One of the speakers replied that at DIMC, the situation is also the same. A lot of students do not plan to become academicians so it is difficult to convince them that they will need English in their future as professionals. However, teachers at DIMC continue to motivate students by constantly reminding them of the advantages and disadvantages of being proficient in English. Moreover, they are also strengthening their study abroad programs based on the positive and promising results from recent returnees and are also increasing sisterhood partnerships with universities worldwide to further facilitate student exchanges. They also emphasize on the need to address globalization and the public's easy access of information through the internet. Patients (even Japanese) have become updated with current information from abroad on medical or dental news. So even if one is in private practice, you will still have to deal with current events

brought up by patients. If someone in private practice does not know this, it will create a bad image because it could mean that that doctor/dentist is not updated. Thus, even if a student plans to go into private practice, he/she has to keep up with the latest information, whether locally or globally. At DIMC, teachers frequently remind students that even in Japanese-language journals, an English abstract is always required.

*Concerning the willingness of clinicians to participate in the two-way video conferencing system to provide clinical content to student discussions*:

At first, it seemed to be a concern among clinicians but once the system became well-established, they became more willing to participate since it is their only chance to interact with students who are learning English terms in his specialty. Clinicians are usually awarded with certificates of appreciation. This will benefit them in building up their CV's (faculty development, curriculum development, international activities).

*Concerning the standardization of students in public and private schools*:

The aim of most schools is to increase the passing rate of board examination passers and because of this; they give priority to board exam courses rather than dental English courses. Thus, some schools do not offer the course, offer them for a few semesters, offer them in the early years of dental school or do not provide study-abroad programs. Since dental English is not a compulsory course, there is a disparity between and among schools regarding these courses. However, standardization is necessary to ensure that students are exposed to the same learning tools. All students (whether in public or private schools) should be given the same opportunities otherwise learning will not be fair. It is also important to make a generalized lesson plan for specified topics so other teachers can just follow it. Since teachers have different backgrounds, it is extremely important to be clear about the objectives and what they want students to learn at the end of the course. Timing is also an important issue. Some schools offer the course only in the first year of dental school when students are not yet even familiar with dental topics in the Japanese context. After this term, there will be no other dental English courses to refresh their memory in the later years when they will have already mastered dentistry in the Japanese context.

Therefore, it is important to offer dental English courses slightly after the standard curriculum so it will serve students well in comprehension and can also serve as a review lesson as well.

*Concerning Prof. Barron's experience in enrolling in English-language training programs for non-native English speakers (at the University of Edinburgh)*

For Prof. Barron, who is a native speaker of English, he still found the two-week course to be valuable.

*Concerning teaching and learning techniques from the perspective of a bilingually-raised Japanese (Ms. Aya Watanabe)*

One of the most important issues to consider in teaching English to Japanese learners is the structure of sentences. Asians put emphasis on understanding the context (readers take the responsibility to understand) while Westerners put emphasis on writing well (writers are responsible for conveying their message to readers). Prof. Barron elaborated by stating an example evident during the review process of publication. Most referees read papers after work or late at night when they are tired. So papers have to be written in a way that is simple for the reader to understand. Writing (learning how to communicate in writing) should be practiced more by Japanese students. Another example stated was that lawsuits in the US are most often based on communication (You said that…, No, I didn't!). If the doctor has a caring attitude, explains well and then makes a mistake, the patient is less likely to sue compared to an arrogant and not communicative doctor.

*Concerning the membership eligibility for JASMEE:*

Japan Society for Medical English Education (JASMEE) welcomes any educator who is interested in any health-related profession. A dental chapter/component will be another driving force in pushing for better English education in the professions.

*Concerning the effectiveness of including a standardized medical/dental English education into the national board examination to motivate students:*

When Professor Barron opened the idea of the possibility of including medical English in the board exams, the Ministry of Employment was against it because the medical licensing exam in Japan is to test whether doctors can treat Japanese patients. Interestingly, in the following years, English questions started to appear in the exams. However, at the JASMEE meetings, it is always emphasized that this is not a medical test but rather a test of language in the field of medicine. JASMEE does not have a special committee for the purpose of including English in the licensure exams. However, if there were to be a greater move towards English questions in the licensure exams, surely that would have a significant effect in motivation.

*Concerning the core curriculum:*

It will be realistic and practical to offer General English for one year and Dental English also for one year (two semesters for each). As I presented for deliberation a one-semester and a two-semester course for dental English, I had to ask everyone about their opinions. Prof. Langham commented that this would really depend on the respective schools because some allot only one semester for dental English while others allot five or more semesters for it. So it is better not to decide exactly how many semesters but instead present a framework or basis for each one (schools) to mix and match. It is important to give schools the flexibility. Of course, the more semesters (in this case, the two-semester), the better. Prof. Harland also commented that we should develop a two-semester dental English course. The core curriculum just has to provide a lot of materials or ideas for the schools to pick or choose in case they only allot one semester for the course. If the school has two or more semesters allotted for the course, they can also expand some areas. We should therefore develop a core with a basic structure otherwise there will be less flexibility. The big advantage of this grant (gathering everyone for a meeting) is to develop a core flexible enough for everyone to bring back to their home institutions with something they can actually use in their respective situation or school setting. We should be

realistic but not too small so a two-semester core should be developed and schools will decide according to their needs. Therefore, two semesters for the core curriculum was agreed upon by all of the participants.

*Concerning the importance of the first meeting to assure a successful course:*

It is important to set the aims and expectations in the first meeting. Inform the students what they are going to study, explain why they need to study it and how they will be assessed (exams, activities, attendance…). Students need to know what is required and what they need to do to pass the course. This is because most of the students don't read the syllabus or tend to forget it. It is also very important to set the rules and provide a concrete course outline.

*Concerning early establishment of self-confidence:*

If possible, ask the students to introduce themselves in a friendly environment during the first meeting (by pairs or by groups). This will give them the idea that the course will have active learning activities as opposed to the typical lecture-based courses. The peer to peer interaction will also help them slowly build confidence in speaking.

## SUMMARY OF THE WORKSHOP

After taking into consideration all possible concerns, the discussions became focused on motivating students to learn how to improve patient and inter-professional communication and proved to be an important step in deciding the General Instructional Objective (GIO) and Specific Behavioral Objectives (SBOs) for the core curriculum. At the concluding part of the workshop, the GIO and SBOs for the two-semester core curriculum were formulated as a group effort. Both will hopefully come as a guide for syllabus developers of dental English courses. A sample syllabus taking into consideration the above-mentioned objectives are presented in the proposed core curriculum document.

**1st semester**

GIO: Students will learn how to communicate with English-speaking patients on basic topics of routine dental consultation and treatment.

SBO 1: Students will be able to say and respond to basic dental terms and phrases.

SBO 2: Students will be able to understand what the patient communicates in English.

SBO 3: Students will be able to ask their patients about their medical and dental conditions as well as explain dental procedures in English.

SBO 4: Students will be able to access and be able to use patient fact sheets for native speakers at websites, e.g., American Dental Association, American Dental Education Association, International Dental Federation, World Health Organization, etc.

**2nd semester**

GIO: Students will acquire the basic skills of professional communication.

SBO 1: Students will be able to say and respond to dental terms and phrases.

SBO 2: Students will be able to create posters on dental topics.

SBO 3: Students will be able to make oral presentations on dental topics.

SBO 4: Students will be able to correspond with other health professionals

(For example: email, lectures, meetings, etc.).

## CLOSING REMARKS (Yoshida)

Dr. Yoshida thanked the speakers and the participants for sharing their time and thoughts and for making the 2nd meeting successful and invited them for the 3rd meeting. She wished everyone a safe trip home.

## INTERIM CONCLUSIONS AND RECOMMENDATIONS

The meeting facilitated the development of a more concrete outlook and progress for the proposed core curriculum. GIOs and SBOs were proposed and approved by the majority of the participants. The participants also approved holding a 3rd and final meeting and further agreed on the importance of the presence of a MEXT representative as suggested by the organizers. The participants however expressed concern about the implementation of the curriculum and what will happen to the group after the grant is completed. Since there are still so many things to be done, a recommendation was adopted to continue procuring grants and developing collaborative relationships between participants and their programs.

## MESSAGES FROM THE PARTICIPANTS

The participants thanked each other for the opportunity to learn and share. Here are some closing comments from the speakers and participants:

**J.P. Barron**: *I encourage everyone to continue supporting Omar's grant and developing dental English education. He was able to do what I failed to do many years ago. I also encourage everyone to attend the JASMEE conference, either as a participant or presenter.*

**Edward Barroga**: *The initial steps in developing a core curriculum through meetings like this are very important. I encourage all of you to continue developing and improving the core. "Systems cannot be perfect… but they can be optimized".*

*Optimization can be achieved by regular assessment and sharing of ideas. I therefore suggest putting up a Yahoo Group or mailing list to facilitate information sharing and scheduling. This will ensure a fast and effective means of communication between the participants for the 3rd meeting.*

**Chieri Noda**: *I congratulate Omar and all the participants of this meeting for the progress made in devising a core curriculum. The collective effort will certainly be a great asset to all those involved in teaching English for dental purposes, but most of all it will be to the benefit of the students.*

**Aya Watanabe**: *I would like to congratulate Omar and everyone for gathering around the table to discuss such an important topic. Deciding on a core curriculum is very difficult but it is such an important task for future dental students as well as the instructors. I have no doubt that these discussions will lead to further advancement of the field of Dental English Education. Thank you everyone for the precious learning opportunity. I look forward to hearing about future development of this project.*

**Martin Peters**: *I agree with the core curriculum. I also found out that motivating students is the perennial and crucial issue common in all of the discussions. Addressing the issue on how to tackle motivation is therefore very important. I am frustrated with the lecture-style arrangements for classes so we have to do some changes to motivate students to learn.*

**Naomi Fukai**: *I am thankful to be given this opportunity to participate. It was indeed a learning experience for me.*

**Roxana Stegaroiu**: *Thank you once again Omar for organizing this meeting. I would like to suggest providing a Japanese translation of the core curriculum and reports. The translations will offer clarity and convenience for teachers, schools and education officials.*

**Kazuyoshi Suzuki**: *Thank you again Omar for organizing the meeting and giving me another learning opportunity.*

**Michiko Nishimura**: *I am not directly involved in dental English education but I was able to learn a lot from the discussions. For example, Jane's blended learning is very much applicable to our tutorial system. As we all have different backgrounds, I think that forums like these provide the best learning experience.*

**Tomiko Yamagami**: *We do not have a definite dental English course at our school but I teach dental English to dental hygienists. After hearing all of the discussions, I am more convinced of the importance of dental English courses.*

**Michael Ishii**: *Thank you. The meeting was very informative.*

**James Hobbs**: *I would like to thank Omar for organizing the meeting, the speakers and everyone here who shared their thoughts. We have now made progress and I also agree that we have to continue keeping in touch with each other.*

**Shigeru Takahashi**: *I am thankful to be invited to participate in this meeting. We do not have the dental English course at Hokkaido University but I think we should have the course in the future.*

**Chie Yanai**: *Thank you Omar for organizing the meeting. I learned a lot again. I also thank the speakers… I met Professor Barron at a conference before and I learned so many ideas concerning motivation from him. I also think that motivating students should be an important part of faculty development and the core curriculum.*

**Masaki Ohno**: *Thank you everyone. Teaching is an important job but it is difficult. This is especially true for Shika Eigo. That's why this meeting is important.*

**Jane Harland**: *Thank you Omar for organizing the meeting. Meeting everyone here is better than getting in touch by email. Meetings make it easier for everyone to share knowledge and opinion. For me, every day is a learning process. Teamwork and communication between the clinical faculty, English teacher and the student is important. Language teachers cannot do everything while the same is true with dentists in teaching dental English courses. Teamwork is therefore important. If we combine our work together, we double the strength. So I hope in the future, there will be more opportunities to collaborate and cooperate with each other.*

**Ikuo Kageyama**: *I would like to thank Omar's grant because without it, none of our meetings, discussions and developing the core curriculum will be possible. So Omar is the key person to all of this. We have come to realize the current situation of dental English and its importance. It is now our responsibility to continue what has been started.*

**Asiri Jayawardena**: *I wanted to attend the 1$^{st}$ meeting but I was not in Japan so now, I am thankful I am here. I have learned so many things from the speakers and all of you. Thank you for the great learning experience.*

**Yoshiaki Shibaie**: *I am very happy that we were able to make concrete progress concerning our core curriculum. I hope we will also be able to come up with a wordlist to include in the core.*

**Etsuko Watanabe**: *Thank you once again for the learning experience. The meeting was great. I suggest that Omar should give us assignments to do next time so that we can contribute to his paperwork.*

**Yo Shibata**: *The lecture brought up an interesting approach of English core curriculum in Japanese Dental School as well as in depth analysis of its efficiency, which might give some insight to future globalization of our Dental school. I do hope to continue conversation with the participants and am interested in attending the JASMEE conference.*

**Yoshinaka Shimizu**: *It is my first time to attend this significant meeting so thank you for the learning experience.*

**Clive Langham:** *This was a very productive meeting in which the curriculum was further discussed and concrete suggestions were put forward. This will really assist people involved in English education at dental schools in Japan and will speed up the movement for introduction of dental English classes.*

**Hironori Tsuchiya**: *I'd like to thank Dr. Omar for organizing this instructive meeting following the first Okayama Forum. Considering diversity in the number of English classes and teachers, the syllabus and the school year of different universities, it's very difficult to make a curriculum common to all dental schools. But it may be possible to develop a fundamental or essential one. Even students lacking the basic achievement and the motivation to be a dentist are entering dental schools, especially in private universities, nowadays. It's urgent to increase students' interest in learning English specific to dentistry after making them understand its significance and importance. I think we should cooperate with JASMEE, which I've participated these past ten years. As all members know, the National Board Dental Exam has set several questions in English since 2010, the 103rd exam. I also think English teachers could at least partly contribute to the preparation for taking the Exam by teaching dental terms and terminology. I'm looking forward to attending the third meeting to share knowledge and experience.*

**Naoko Seki**: *Thank you so much for the informative meeting. I learned many things from the speakers and all the participants. Thank you.*

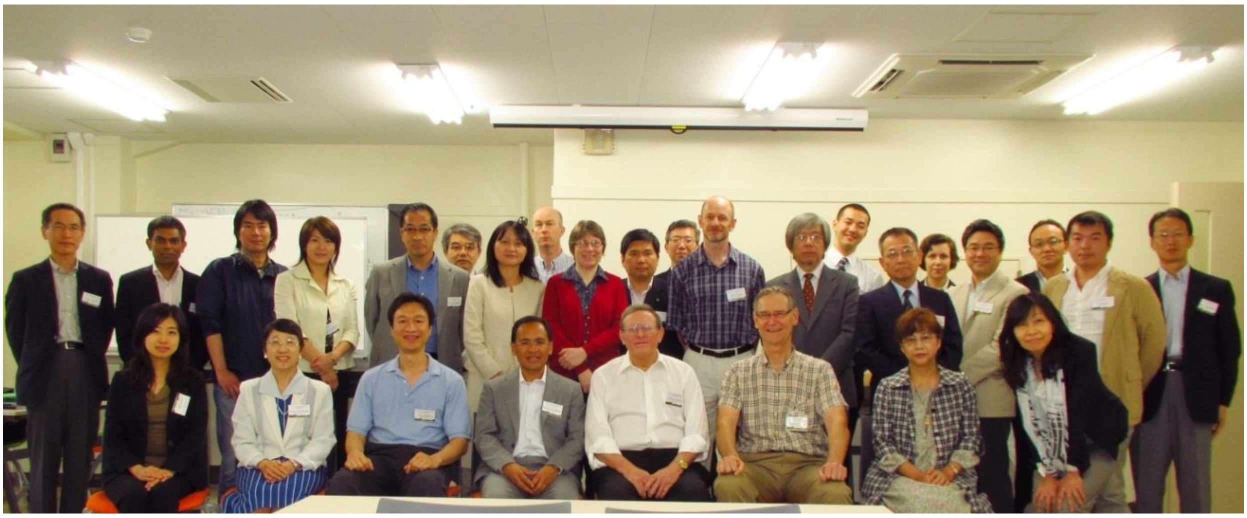

**Back Row** (L to R): Yoshiaki Shibaie, Asiri Jayawardena, Masaki Ohno, Etsuko Watanabe, Ikuo Kageyama, Naoyuki Kariya, Chie Yanai, Clive Langham, Jane Harland, Yujiro Handa, Yoshinori Sahara, James Hobbs, Naomi Fukai, Michael Ishii, Hironori Tsuchiya, Roxana Stegaroiu, Kazuyoshi Suzuki, Yoshinaka Shimizu, Yo Shibata and Shigeru Takahashi
**Front Row** (L to R): Naoko Seki, Tomiko Yamagami, Omar Rodis, Edward Barroga, J. Patrick Barron, Martin Peters, Michiko Nishimura and Toshiko Yoshida. **Not in Photo**: Chieri Noda, Aya Watanabe, Hiroko Oka and Junichi Fujita

*On behalf of my co-investigators, I would like to extend my sincerest thanks and appreciation to the speakers, participants and guests of the 2nd meeting held in Tokyo. Your presence, support, interest and valuable comments and suggestions made our meeting successful. I look forward to seeing you again in our future meetings. I would also like to express my sincere appreciation to Dr. Naoko Seki, Professor Ikuko Morio and the administrative staff of Tokyo Medical and Dental University, for their assistance in facilitating the use of their conference rooms as our venue.*

Omar M.M. Rodis
